# Inflammatory Pathways in Overweight and Obese Persons as a Potential Mechanism for Cognitive Impairment and Earlier Onset Alzeihmer’s Dementia in the General Population: A Narrative Review

**DOI:** 10.3390/biomedicines11123233

**Published:** 2023-12-06

**Authors:** Alexandru Dan Costache, Bogdan Emilian Ignat, Cristina Grosu, Alexandra Mastaleru, Irina Abdulan, Andra Oancea, Mihai Roca, Maria Magdalena Leon, Minerva Codruta Badescu, Stefana Luca, Alexandru Raul Jigoranu, Adriana Chetran, Ovidiu Mitu, Irina Iuliana Costache, Florin Mitu

**Affiliations:** 1 Faculty of Medicine, University of Medicine and Pharmacy “Grigore T. Popa”, 700115 Iasi, Romania; adcostache@yahoo.com (A.D.C.); alexandra.mastaleru@gmail.com (A.M.); irina.abdulan@yahoo.com (I.A.); andra.radulescu@yahoo.com (A.O.); roca2m@yahoo.com (M.R.); leon_mariamagdalena@yahoo.com (M.M.L.); minerva.badescu@umfiasi.ro (M.C.B.); stefana.luca08@gmail.com (S.L.); jigoranu.alexandru@yahoo.ro (A.R.J.); adriana.ion@hotmail.com (A.C.); mituovidiu@yahoo.co.uk (O.M.); ii.costache@yahoo.com (I.I.C.); mitu.florin@yahoo.com (F.M.); 2 Clinical Rehabilitation Hospital, 700661 Iasi, Romania; 3 “St. Spiridon” Emergency County Hospital, 700111 Iasi, Romania; 4 Romanian Academy of Medical Sciences, 927180 Bucharest, Romania; 5 Romanian Academy of Scientists, 050044 Bucharest, Romania

**Keywords:** obesity, overweight status, cognitive dysfunction, dementia, Alzheimer’s disease, older population, body mass index, waist-to-hip ratio, inflammation, lifestyle intervention, dietary measures

## Abstract

The overweight status or obesity can be confirmed through classical methods such as the body mass index (BMI) and the waist-to-hip ratio (WHR). Apart from metabolic issues such as atherosclerosis, liver steatosis, or diabetes mellitus, long-term obesity or overweight status can pose a risk for cardiovascular and neurovascular complications. While some acute adverse events like coronary syndromes of strokes are well-documented to be linked to an increased body mass, there are also chronic processes that, due to their silent onset and evolution, are underdiagnosed and not as thoroughly studied. Through this review, we aimed to collect all relevant data with regard to the long-term impact of obesity on cognitive function in all ages and its correlation with an earlier onset of dementia such as Alzheimer’s disease (AD). The exact mechanisms through which a decline in cognitive functions occurs in overweight or obese persons are still being discussed. A combination of factors has been acknowledged as potential triggers, such as a sedentary lifestyle and stress, as well as a genetic predisposition, for example, the apolipoprotein E (ApoE) alleles in AD. Most research highlights the impact of vascular dysfunction and systemic inflammation on the nervous system in patients with obesity and the subsequent neurological changes. Obesity during the early to mid-ages leads to an earlier onset of cognitive dysfunction in various forms. Also, lifestyle intervention can reverse cognitive dysfunction, especially dieting, to encourage weight loss.

## 1. Introduction

One of the most significant public health problems is obesity. Cognitive decline is linked to excess adipose tissue, especially when it is distributed centrally. Indeed, obesity has been linked to several negative changes in the brain’s structure and function that can be identified using neuroimaging methods. These alterations caused by obesity might cause cognitive dysfunction [1].

Classically, obesity has been described as an increased body mass index (BMI) starting from the value of 30 kg/m^2^. While it is a parameter still in use today and is easy to apply in any clinical setting, it does not offer enough information to quantify the metabolical risk of patients (e.g., persons with a high muscle tissue percentage who have an increased weight but a reduced percentage of adipose tissue). Therefore, it is recommended to be used in conjunction with another anthropometric measurement, the waist-to-hip ratio (WHR). Nowadays, the definition of obesity involves both the BMI value above 30 kg/m^2^ and the WHR. WHR is also a major criterion for the metabolic syndrome based on the harmonizing definition [2,3].

The average age of the population in Europe is increasing, resulting in a higher prevalence of comorbidities that require additional care. Aging is frequently associated with a decline in cognitive functions and is a high-risk factor for impairing conditions like Alzheimer’s disease [4,5,6,7,8]. The onset and severity of dementia are influenced by genetic factors, particularly in relation to the age at which it develops and the extent of cognitive decline. ApoE is also strongly involved in Alzheimer’s disease (AD) with its alleles (*ε*2, *ε*3, and *ε*4). Depending on the type of expressed alleles, the onset of the disease may vary. ApoE *ε*2 is often associated with the onset of dementia occurring later in life, while ApoE *ε*4 is typically associated with an earlier onset of the disease and a more severe form of dementia progressing over time [9]. If the genetic predisposition is present, there are several other factors that may contribute to an additive process in a patient with the ApoE *ε*4 allele. Impaired baseline creatinine clearance and chronic significant alcohol intake are detrimental to early cognitive and functional alterations. On the other hand, constant school attendance and education had a protective effect on those individuals with the ApoE *ε*4 allele. Similarly, a history of head trauma with loss of consciousness was dangerous for early cognitive deficit onset in these individuals, whereas lifelong correctly conducted hygiene measures were protective for later cognitive outcomes [10].

While several pathogenic mechanisms have been elucidated, definitive curative therapy is still eluding, and the only feasible measures are those targeting risk factors such as obesity [11,12,13,14]. In Europe and North America, the rates are increasing, and the population is suffering from its complications, i.e., impaired glucose tolerance and the subsequent type 2 diabetes mellitus, as well as systemic inflammation. Metabolic syndrome has been shown to be linked with both short- and long-term cognitive decline [15,16,17,18,19].

The involvement of the obese or overweight status and the onset of any form of cognitive impairment has been highlighted by several studies already, and newer research is also acknowledging this aspect. Gong et al. conducted a study involving 5809 individuals aged 60 and above, focusing on changes in BMI. Their findings suggest that transitioning from non-obese to obese and attaining peak weight between 18 and 40 years old is linked to cognitive alterations in later stages of life [20]. Similarly, Chuang et al. evaluated a cohort of 618 persons aged over 60 years with an emphasis on a personal history of high blood pressure values and an increased waist circumference at any point in their life and were also given the Mini-Mental State Examination (MMSE) for their cognitive function assessment and the hand grip strength and 4 m walking speed for physical capacity evaluation at the present moment. They concluded that higher values for waist circumference or for blood pressure in midlife were associated with a decline in both physical ability and cognitive function in later life. Yet, those who underwent measures to correct the increased body mass and to control their blood pressure values showed a reduction in mental and physical decline [21].

One interesting retrospective study was conducted by Twig et al. on 2,277,188 individuals from Israel who were aged between 16 and 19 years and were in the premilitary recruitment phase. They all underwent general intelligence tests, and with the aid of Cox models, the correlations between cognitive function and different causes of mortality were tested. Their cognitive functions during their younger years were correlated with the risk for diabetes, cardiovascular-related mortality, and all-cause mortality in their later stages of life [22].

The importance of this subject is also sustained by the fact that metabolic complications, such as higher obesity rates, metabolic syndrome, diabetes, nonalcoholic fatty liver disease, and neurobehavioral disorders, such as attention deficits and hyperactivity, have been observed in children of mothers diagnosed with type 1 diabetes mellitus. Interestingly, neurobehavioral disorders were more frequent in male offspring, while metabolic complications were more evident in female offspring [23].

Interestingly, Cukierman-Yaffe et al. have shown an independent association between an elevated risk for impaired fasting glucose regardless of gender and lower cognition in late adolescence in a retrospective cohort study [24].

While studying the mechanism and the long-term consequences, several studies are directed toward preventive or curative measures. Hence, a significant emphasis is placed on examining how dietary intervention or bariatric surgery can contribute to mitigating the decline in cognitive function or potentially reversing it [25].

However, Boidin et al. conducted a study on 54 obese and 16 non-obese individuals in 2020. The obese group was divided into high-fitness and low-fitness individuals based on their median aerobic fitness, which was divided by lean body mass. They were subjected to several cognitive tests assessing short-term and working memory, processing speed, executive function, and long-term verbal memory. The conclusion was that high-fitness obese individuals showed better short-term memory and executive function performances in comparison to low-fitness obese persons. This could emphasize the fact that a higher fitness level may be protective of cognition, regardless of an obese status diagnosis [26].

Therefore, this narrative review aims to gather relevant data published regarding the correlations between cognitive impairment in individuals and the association of the obese or overweight status and offer insight into the pathophysiological mechanisms involved and potential indications for the inhibition or reversal of such processes.

## 2. Pathophysiological Mechanism of Cognitive Impairment in Obese Persons

Cognition is a global concept encompassing various processes virtually scattered over the whole brain. Obesity itself can be viewed as a consequence of impaired energetic feedback loops or as a higher disorder of impaired reward—behavior control mechanisms. However, the adipose tissue—brain relation is much more than a simple direct bilateral communication involving virtually all metabolically active organs and a plethora of messengers. Although most of the research has focused on the hypothalamus and the hippocampus, wider abnormalities in the central nervous system were found to correlate with obesity. White matter diffuse damage and focal white matter abnormalities [27], connectivity loss [28], and cortical and subcortical gray matter atrophy [29,30,31] were found in human imagistic studies in obese subjects, and the pattern of grey matter atrophy is similar to the one found in neurodegenerative diseases [32]. As multiple factors influence results, sometimes correlations can appear conflicting—e.g., obesity correlates with abnormal brain perfusion in subjects with normal cognition and mild cognitive impairment but not in subjects with Alzheimer’s disease. In the same study, white matter integrity in the corpus callosum, superior longitudinal fasciculus, inferior fronto-occipital fasciculus, fornix, and cingulum negatively correlated with BMI in the cognitively healthy sample but not in subjects with mild cognitive impairment (MCI) or Alzheimer’s, while gray matter’s volume negative associations with obesity were more extensive in the cognitively healthy group than in the MCI group [33].

The direct mechanism and causality of obesity in relation to neuronal structural, functional, and cognitive parameters is still unclear, and whether association implies causality remains to be established. Abnormal impulse control and obesity share anomalies in motivation and reward-related brain centers. The generally accepted mechanisms for obesity-related CNS dysfunction are inflammation [34], vascular abnormalities (with endothelial dysfunction as an essential actor) [35,36], and, in a less definite manner, neuronal energy homeostasis (due to mitochondrial dysfunctions [37]) or direct influence of adipose tissue signals in the brain. Nutritional factors that are less specifically tied to these processes could also play significant parts—minerals such as zinc in particular, with zinc supplementation improving cognitive performances in obese women independently from weight loss [38]. Aside from “traditional” cellular and soluble factors, adipose tissue can secrete extracellular vesicles that can serve as mediators to communicate with other peripheral tissues, such as the liver and skeletal muscles. In a recently published study, the authors show that specific miRNAs are significantly upregulated in participants with obesity and diabetes and can be transferred via extracellular vesicles from the adipose tissue to the hippocampus. Among upregulated miRNAs, miR-9-3p showed a coherent upregulation trend in the hippocampus of mice fed with a high-fat diet and in the extracellular vesicles of humans with diabetes, inducing synaptic damage and cognitive impairment in both [39].

In obese persons, adipose tissue initiates a local immune response by activating multiple immune cells. An increased volume of adipocytes may be the first step in attracting macrophages and triggering their activation (shift to M1 state), beginning local immune accumulation [40]. The activated immune cells then may interact with CNS immune system cells or glial cells through various transmitters or may circulate and pass through the blood–brain barrier (BBB), maintaining a global inflammatory state. Systemic inflammation echoes in the brain, at least partly due to BBB dysfunction [37,41] and abnormal activation of CNS residents and peripheral immune system components. CNS inflammation is associated with functional and structural abnormalities of the brain. A high-fat diet induces memory impairments in mice as soon as three days after the initiation and depression-like behavior after five days, possibly explained by very prompt BBB dysfunction from the first day. However, in the same study, BBB permeability returned to normal at two weeks and increased again after four weeks—supporting the conclusion that various mechanisms contribute to cognitive abnormalities and that CNS aggression is most likely multifaceted [37].

Decreased neuronal and increased non-neuronal cell numbers and densities in the hippocampus and increased non-neuronal cells in the frontal cortex and hypothalamus of obese mice suggest a tight connection between inflammation and neurodegeneration [42]. Dystrophic hypothalamic microglia (and, to a lesser extent, astroglia) were found in obese brains when compared to normal-weight individuals [43].

Adipose tissue also produces messengers that exert their effects remotely. Leptin, a peptidic hormone synthesized mainly by white adipose tissue, appears to have various effects on central nervous system structures, mostly related to energy regulation. Normal neurodevelopment of hypothalamic structures such as the arcuate nucleus seems to require specific patterns of leptin intervention in terms of timing and duration, with potential enduring consequences in the case of abnormal stimulation [44]. Leptin is increased in obesity [45]. Its effects in the brain depend mainly on leptin receptors found not only in the hypothalamus but also in the hippocampus, amygdala, and cerebellum. In the hippocampus, leptin facilitates synaptic efficacy of both excitatory and inhibitory synapses, potentially inducing either long-term potentiation or long-term depression and is likely to have procognitive actions related to learning and memory processes and memory consolidation [46]. Leptin may have protective actions against the acute and chronic synapto-toxic effects of amyloid β, promote amyloid β clearance, and enhance neurogenesis [46]. In a populational study, higher levels of leptin and resistin (but not adiponectin) were associated with a reduced risk of dementia in overweight/obese persons and not in persons with a BMI of 25 kg/m^2^ [47]. In another study, leptin levels were significantly higher and adiponectin significantly lower in obese patients. The same research has shown that while high levels of adiponectin were associated with neurodegenerative dementia and high levels of resistin with vascular dementia, leptin levels did not differ significantly in demented patients as compared to normal subjects [48]. Decreased sensitivity to leptin (“leptin resistance”) may be due to receptor abnormalities, impaired transport across the BBB, or increased activity of its negative regulators [49]. Obesity might be caused by chronic metabolic inflammation in the hypothalamus, possibly as a direct neuronal response to chronic overnutrition (local oversupply of glucose or lipids) [50]. In another neurotoxicity paradigm, leptin can inhibit neural stem cell expansion by specific receptor-mediated (via ERK/cyclin D1 pathway) apoptosis of neuronal precursors in vitro models. Whether cognitive impairment (and possibly Alzheimer’s disease) associated with obesity is due to leptin resistance (similar to cognitive deficits in leptin-deficient models) is still an uncertainty [51].

In a similar manner, abnormal gut microbiota associated with obesity appears on one side to generate inappropriate activation of the immune system and on the other side to release in circulation substances that can pathologically interact with the BBB and the CNS immune system (of whom lipopolysaccharide (LPS) is probably the most researched) [52,53]. Obesity due to a high-fat diet in wild mice was associated with gut dysbiosis—decreased relative abundance of *Faecalibaculum* and increased relative abundance of *Dubosiella* [54]. The authors found increased cognitive deficits and white matter lesions in obese mice subjected to reduced cerebral flow and attributed these to increased inflammation triggered by lipopolysaccharide (LPS) through a TLR4-dependent mechanism. Intestinal permeability, plasma LPS levels, and levels of proinflammatory cytokines IL-6 and IL-1β were significantly greater among HFD mice. In the corpus callosum, significantly higher LPS levels, TLR4 expression, activated microglia, reactive astrocytes, oxidative stress, and BBB permeability markers were found, while tight junction protein occludin was downregulated [54]. In another study in humans, obesity was associated with an increase in the *Prevotella*/*Bacteroides* (P/B) ratio as well as with an increased centrality of the nucleus accumbens (reflecting the ability to affect the signal of connected structures) and a decreased centrality of brainstem structures involved in food intake regulation, as well as a decrease in fecal tryptophan [55]. In another study, the scores of all memory domains were associated with altered plasma levels of tryptophan, tyrosine, and phenylalanine and their catabolites, but memory-related alterations in tryptophan metabolism were only observed in individuals with obesity [56]. In the same study, common species that positively associated with learning, verbal memory, and working memory belong to the *Firmicutes phylum*, while negative associations between the gut microbiota and memory scores were identified within the *Bacteroides* and *Proteobacteria* [56]. Quinolinic acid levels were significantly increased in obese compared to non-obese humans and significantly correlated with BMI [57]. Quinolinic acid is a metabolite of tryptophan, acts as a (neurotoxic) glutamate receptor agonist, and is excessively secreted by activated macrophages and adipose tissue during inflammation [58]. In humans, serum quinolinic acid levels were negatively correlated with the total cognition score of the Repeatable Battery for the Assessment of Neuropsychological Status, particularly with regard to the delayed memory index, while in obese mice, they were negatively associated with recognition memory and spatial working memory [57].

Another possible mechanism for gut microbiome to connect to immune disorders and cognitive abnormalities involves short-chain fatty acids (SCFAs) (mainly acetate, propionate, and butyrate) produced by gut bacteria from nondigestible dietary fibers. These are major cellular energy sources and appear to modulate the strength of the gut barrier, promote cellular metabolism, and have significant immunomodulatory activity [59]. Long-time early supplementation of SCFAs in murine and cellular models for neurodegenerative diseases increased neuronal resistance to oxidative damage, enhanced astrocyte–neuron communication, and alleviated the cognitive impairment by reducing Aβ deposition and tau hyperphosphorylation [60]. However, the neuroprotective effect of SCFAs remains to be clarified—i.e., in another mouse model for Alzheimer’s disease. The administration of SCFAs increases microglial reactivity and activation and increases Aβ plaque loads [61]. The correlation between gut microbiota and obesity is inconstant in human studies, with a meta-analysis showing obese adults to have higher feces levels of SCFA but no statistically significant differences between *Bacteroides* and *Firmicutes phyla* [62]. Supplementation of butyrate in obese mice improved metabolic control and cognitive parameters, generating a number of neuroprotective effects in the frontal cortex of treated mice. Its effects were probably mediated through an increased expression of BDNF in the frontal cortex due to epigenetic regulation, thus preventing a decrease in total neurite length, number of neurite branches, dendritic arborization and complexity, and have protective effects on synaptic spine morphology and spine density [57].

Information from the gut through the vagal afferences can induce behavioral changes (promoting hippocampal-depending learning and memory processes), and gastrointestinal vagal disconnection leads to impaired memory and decreased neurotrophic (BNDF) and neurogenesis markers (doublecortin) in the hippocampus [63]. Similarly, a high-fat diet can induce by itself changes in hypothalamic microglia (both pro- and anti-inflammatory), and these changes preclude the increase in pro-inflammatory cytokine expression (supporting a direct response of microglia to diet) and appear to downregulate genes involved in sensing microenvironmental alterations. Whether this reflects that obesity and gut microbiota changes result from a specific obesogenic neural activity or whether the central pattern of activity is the result of obesity and intestinal changes remains to be established [43].

## 3. Cognitive Ability Domains

If we classify cognitive ability domains by the general process involved (sensation, perception, motor skills, attention and concentration, memory, executive function, processing skills, and verbal skills), memory and executive functioning are probably the most studied in relation to obesity. At the opposite end are motor and language/verbal skills, which are evaluated in a minority of studies [64]. The authors found patients with metabolic syndrome to perform worse in phonetic and semantic fluency tasks compared to normal subjects but found no correlation with waist circumference (although other components of the metabolic syndrome correlated with verbal performance) [65]. Phonemic verbal fluency (as well as delayed recall) was associated with abdominal obesity in another study on metabolic syndrome patients, while immediate recall and attention, mental flexibility, and visual–motor skills (evaluated by the Trial Making Test) were not [66]. Similarly, significantly lower phonemic fluency and lower inhibitory control were observed in obese patients without eating disorders as compared to healthy-weight controls [67].

Motor skills are included more frequently in children’s studies, with obese or overweight preschoolers showing worse fundamental/gross locomotor skills as compared to eutrophic children in some studies, while others did not highlight it [68,69,70].

While global cognitive impairment is constantly demonstrated in obese persons, cognitive domain analysis does not always show correlations. Salama et al. show that although mild cognitive impairment is significantly more frequent in obese persons (with a crude odds ratio of around 5), both the total score and the five cognitive domains of Addenbroke’s Cognitive Examination (ACE) fail to correlate with the BMI. However, in the same study, there was a significant difference between normal subjects and grade II and III obese subjects in fluency and memory scores, as well as in the total ACE score [71].

Suboptimal executive functions, impaired decision-making, and impulsivity are frequently associated with addictions—of particular interest for us is food addiction [72]. Obese patients with cardiovascular disease showed a lower level of sensitivity to cognitive interference, lower abilities in divided attention during visual-tracking tasks, and greater impulsivity than normal-weight cardiovascular patients (as manifestations of attentional and executive dyscontrol) [73]. In another study, obese patients showed impaired performance on the decision-making task (Iowa gambling task) and performed the worst on the set-shifting task (Wisconsin Card Sorting Test). Moreover, trend analysis across Iowa gambling task blocks within each group revealed that the obese sample did not learn during the task [74]. In a meta-analysis that included 72 studies—with 4904 overweight/obese participants—the authors found broad impairments of executive function (including on tasks primarily utilizing inhibition, cognitive flexibility, working memory, decision-making, verbal fluency, and planning) to be associated with obesity, while overweight participants only showed significant deficits in inhibition and working memory [75]. 

In a recent study on 474 healthcare professionals, the authors used the self-reported Gilewski questionnaire for a global assessment of memory complaints [76]. Significant differences were observed in the level of general frequency of forgetting, the seriousness of forgetting, retrospective functioning, and mnemonics usage in relation to different body mass indexes and body fat percentage classes (*p* < 0.05). The findings showed a positive correlation between excessive body fat and general frequency of forgetting and seriousness of forgetting, whereas a negative correlation was observed to retrospective functioning and mnemonics usage [77]. In terms of memory subdomains, many studies found obese persons to suffer from working memory deficits [75,78,79]. Higher BMI was also associated with nonverbal (visuospatial) learning deficits in individuals with lower education (with higher education having a protective effect against the effects of obesity) [80]. Episodic memory was also reported to be affected by obesity in some studies, while in others, although overall memory ability was similar, there were clear differences in the use of learning strategies (with normal adults using semantic clustering and serial clustering more effectively than adults with obesity during all test phases) [81,82].

Cognitive impairment in obese persons covers a wide range of cognitive domains. The exact contribution and the possible causality relation between them is far from understood and remains a matter of debate as different studies with conflicting results (at least in appearance) continue to emerge.

## 4. Consequences Observed on Obese Patients from a Neurological Perspective

Obesity in midlife is a major risk factor for Alzheimer’s disease (AD) and vascular dementia (VAD) later in life. There is evidence that obesity not only increases the risk of early cognitive decline but also can have an immediate negative impact on cognitive function. There is a negative correlation between anthropometric measurements of obesity and various cognitive domains. For example, obesity is associated with decreased performance on tasks related to episodic memory, and language learning is impaired in people with high or low BMI. Similar deficits are observed in visual modality (VOM) memory tasks. Additionally, reduced working memory performance is observed in obese and overweight young adults compared to healthy controls. Furthermore, performance impairments are also evident in other cognitive domains unrelated to memory. However, this is not consistently observed in obese cohorts. Decreased performance in executive functions (such as concept formation and sentence switching) is also observed in obese cohorts compared to normal-weight controls [83,84,85,86,87,88,89,90,91,92,93,94,95].

For the diagnosis of mild cognitive impairment (MCI), the MCI Working Group of the European Consortium on Alzheimer’s Disease, Brescia Meeting, Italy, June 2005 Criteria are used (see Table 1) [96,97].

Research has shown that obesity is linked to impaired decision-making abilities in populations with eating disorders, such as anorexia nervosa or bulimia. Moreover, several studies have demonstrated that decision-making is also negatively impacted in obese subjects. The Iowa gambling task suggests that obese individuals have a decreased ability to maximize immediate gratification or program delayed gratification. Additionally, obese individuals demonstrate impaired performance on other tasks that require delayed gratification. The poor decision-making processing of intertemporal choices may play a role in the dietary preferences of obese individuals. This is evidenced by an increased sensitivity to the immediate reward of consuming calorie-dense, energy-dense foods while ignoring the long-term health and metabolic consequences. This suggests that their tendency to prioritize short-term satisfaction over long-term well-being may contribute to their poor eating habits. However, no consistent association between obesity and cognition has been found within or across cognitive domains. This discrepancy may be due to the possible moderating effects of many obesity-related comorbidities, which are known to negatively impact cognitive performance [98,99,100,101].

In some people, type 2 diabetes mellitus (T2DM), high blood pressure, hypercholesterolemia, and insulin resistance coexist; therefore, the results of two recent systematic reviews on the effects of obesity on cognitive performance in adults were inconclusive. The two studies concluded that there is evidence of cognitive impairment in obese populations. However, there was insufficient evidence to support the association of these impairments with co-occurring obesity-related disorders and demographic variables such as age and education, mainly due to the fact that many studies did not adequately control for potential comorbidities [102,103].

One possible pathophysiological pathway was illustrated by Miller et al. Increased levels of circulating free fatty acids and systemic inflammation are caused by obesity and high-fat diets. At the hypothalamic level, circulating cytokines, free fatty acids, and immune cells enter the brain and start local inflammation, including microglial growth. This can lead to alterations in the internal circuitry of the hypothalamus as well as its outputs to other brain regions. This inflammation is also believed to contribute to synaptic remodeling and neurodegeneration, further exacerbating the negative effects on brain function and cognitive health. As a result, brain areas like the hippocampus, amygdala, and reward-processing centers disturb cognitive function. Therefore, central inflammation in obesity has detrimental effects on cognition, disrupting hypothalamic satiety signals and sustaining overeating [104].

Brain imaging studies have indicated a link between obesity and neural atrophy. Obesity has been associated with structural alterations to the brain’s neural architecture. Research has shown that, regardless of age or disease status, an increase in BMI correlates to a decreased brain volume. Additionally, higher BMI is associated with a reduction in grey matter in the temporo-frontal, occipital, and hippocampus cortices, as well as a decrease in white matter integrity throughout the brain. It should be noted that structural impairments may not always be attributed to obesity, as they may be caused by the effects of aging or other obesity-linked conditions (e.g., diabetes mellitus, atherosclerosis, and hypertension). Nevertheless, obesity poses a risk to neural integrity, as it may lead to its impairment. Furthermore, neural imaging studies have revealed altered functional activity. For example, in healthy adults with an elevated BMI, there is a decrease in regional blood flow into the prefrontal cortex. Furthermore, there is a significantly reduced functional activity in the areas of the brain related to episodic memory (hippocampus, angular gyrus, and DORP) in obese individuals. Additionally, a lower working memory task-related activation of the right parietal cortex has been observed in obese individuals [105,106,107,108,109].

The combination of neuroimaging and neuropsychological brain stimulation provides synergistic benefits. Neuroimaging provides a comprehensive understanding of brain circuits and functions, shedding light on the complex mechanisms underlying neuropsychiatric disorders. At the same time, brain stimulation allows for targeted interventions by manipulating specific regions or networks of the brain involved in these disorders. The integration of neuroimaging and brain stimulation helps elucidate the neural basis of neuropsychiatric disorders and optimize treatment strategies. This powerful combination accelerates the development of symptomatic therapies by identifying the neural circuits involved and guiding precise therapeutic interventions. It has the potential to revolutionize bioelectronic medicine, paving the way for personalized treatments and transformative advances in neuropsychiatric care [110].

Research has shown that obesity is linked to an accelerated brain aging process, specifically in terms of white matter atrophy. Middle-aged individuals with obesity appear to experience it the most, which can result in an estimated increase in brain age by approximately ten years. This age group may be particularly vulnerable to developing obesity compared to later stages of life. Additionally, it is believed that white matter atrophy begins to occur in middle-aged individuals. One possible explanation for this is the action of pro-inflammatory cytokines. Adipokines, which are secreted by adipose tissue, can have both pro-inflammatory and anti-inflammatory effects. Adiponectin, a molecule known for its anti-inflammatory properties and its role in energy metabolism and inhibiting cell proliferation, is reduced in the case of obesity. Conversely, chronic inflammatory states and metabolic disorders are stimulated by the upregulation of other adipokines. These changes contribute to the development of white matter abnormalities, as they create a microenvironment that supports such alterations in the brain [111,112,113,114,115,116,117].

It is increasingly clear that elevated BMI and central obesity in midlife are risk factors for later cognitive impairment and have been identified as modifiable risk factors for cognitive decline. However, it appears that obesity may offer some protection against subsequent cognitive decline. Some studies have shown that obese people lose weight more slowly than people of normal weight. The relationship between obesity and subsequent risk of dementia is unclear. The relative risk ratio for AD and vascular dementia (VD) is three to five percent later in life, according to data from a study of obese patients hospitalized between the ages of 30 and 39 years, and the relative risk gradually decreased until 70. However, the study found that people who were overweight at the time of admission were still at higher risk of later developing dementia than those of normal weight. Additionally, people who were obese after 80 years were less likely to develop vascular dementia in advanced ages. It is estimated that people with an increasing BMI between the ages of 40 and 45 have a 74% higher risk of developing dementia later in life [118,119,120,121,122].

Obesity in itself poses a lower risk of dementia in comparison to diabetes, hypertension, and dyslipidemia in both mid and late-life individuals. However, the risk is higher for obesity in midlife compared to later life (2.0 vs. 0.8). A recent meta-analysis of longitudinal studies examining midlife BMI and risk of dementia in later life found that obesity, but not overweight, in midlife was associated with an increased risk of dementia in later life. A review of empirical studies found that the association between dementia risk and obesity is most similar when obesity is assessed in midlife and cognition in late life, with long-term follow-up. However, it is essential to note that the association between dementia and obesity varies depending on the degree of obesity, the outcome of interest, and the age at which a person is classified as obese [123,124,125,126].

However, BMI is not a reliable measure of adiposity as it does not differentiate between muscle and adipose tissue. Longitudinal studies offer a valuable opportunity to track groups of individuals over an extended period, enabling the observation of disease progression and the identification of patterns, associations, and potential factors contributing to age-related changes. One study followed participants aged 65 and older for five years to assess possible links between age-related obesity, weight change, and dementia. The results were adjusted to account for sample demographics and APOE *ε*4. A BMI between 26.3 and 29.6 kg/m^2^ was associated with a lower risk of dementia, including Alzheimer’s disease, compared to a BMI less than 23.4 kg/m^2^. However, age did play a role in the relationship between BMI and waist circumference, with those with a waist circumference > 97 cm being more likely to develop dementia related to stroke. In individuals under 76 years, those with a BMI ranging from 23.4 to 29.6 kg/m^2^ were less likely to develop dementia than those with a BMI outside this range. Dementia risk decreased with increasing BMI in individuals over the age of 76 years. Nevertheless, individuals under the age of 76 who had a waist circumference greater than 97 cm showed a positive correlation with a higher risk of both dementia and Alzheimer’s disease. However, this correlation diminished in individuals over the age of 76. Additionally, weight loss was linked to an increased risk of dementia compared to those who maintained a consistent weight. On the other hand, people who gained weight had an increased risk of stroke-related dementia [127,128,129,130,131].

Another study compared the impact of obesity on performance on global test standards in people aged between 19 and 93. Participants were evaluated every 2–3 years. When measuring global cognitive function, people with higher BMI, larger waist circumference, and higher WHR performed worse than those with lower BMI, smaller waist circumference, and lower WHR. As with all three measures of obesity, decreased performance was associated with increasing age. Similarly, decreased visual memory performance was associated with increasing obesity and age. Language function was also associated with obesity, and the association varied depending on the measure used but did not change with age. Executive function was assessed using two trail-making tests, A and B. A tested mental flexibility and processing speed, and B tested visual search. In A, higher BMI and larger waist circumference were associated with faster performance, whereas in B, higher WHR was associated with slower performance with increasing age. Specifically, BMI, waist circumference, and WHR were associated with poorer performance on both letter and category skills. However, WHR is only related to visuospatial ability with increasing age, with higher WHR slowing the decline. A similar association has been found between weight change and cognitive performance. A second prospective study was conducted to calculate BMI across adulthood (early adulthood, early middle age, and late middle age) and assessed executive function, memory, and mini-MES performance in late middle age. Cumulative obesity was associated with poorer performance on the mini-MES test and tasks related to reasoning and language fluency (phonemic and semantic) compared to normal-weight individuals. The negative impact on mini-MES performance remained even after accounting for health behaviors and measures assessed in late midlife. However, it is important to note that cognitive function is also affected by the cumulative effects of being underweight. Furthermore, being underweight was associated with poorer rational induction, verbal fluency, and performance in the mini-MES. Low body weight and weight loss may be a sign of preclinical dementia, which is characterized by decreased food intake and lifestyle changes. The concept that preclinical weight loss is promoted by cognitive impairment was supported by a review of 19 cohort studies [132,133,134,135].

On the other hand, it has recently been observed that being underweight during middle and old age is associated with a higher risk of dementia. As the BMI increases, the incidence of dementia decreases. Moreover, obese adults in their adult life were found to be 29% less likely to develop dementia than those at healthy weight. Current obesity levels in older adults are inversely related to dementia. This may be an example of the obesity paradox, in which the onset of dementia may be preceded by weight loss in later life and occurs before any signs of cognitive impairment appear. It is supported by a retrospective cohort study of around two million people aged 40 and over in the UK, which suggested that being underweight in middle age and older age increases the risk of dementia. However, this claim has been met with some controversy, as it may be due to the prevalence of general practitioners’ underdiagnosis of dementia at the time of data collection, as well as the adjustment for a variety of factors, including the competing risk of mortality and selection bias, as well as bias in dementia diagnosis in those with a lower BMI/age. All in all, these findings may provide an explanation for the association between late-life obesity and dementia. However, there is limited evidence to support this hypothesis. Possible reasons for the association include increased glucose metabolism due to increased leg muscle mass. This may prevent the pathogenic effects of increased glucose availability due to glucose uptake into muscle [136,137,138,139].

## 5. Stress as an Additive Risk Factor

Stress occurs when an individual experiences a disconnection between their physiological response to a stressful stimulus and their capacity to manage the situation. From a homeostatic point of view, the demand exceeds the organism’s regulatory capacity. Stress responses are primarily mediated by the sympathetic and hypothalamic–pituitary–adrenal (HPA) endocrine systems. The activation of these systems leads to the release of cortisol (derived from the sympathetic and medullary endocrine systems), which in turn induces adaptive survival responses in response to the stressor’s demands. Adaptive, long-term, over-, or repetitive response activation can have a cumulative effect on the organism, leading to an acceleration in the wear and tear of the body’s systems. Chronic psychological stress leads to physiological dysregulation, which has been linked to multiple and widespread adverse health and life outcomes, including quality of life and longevity [140,141,142,143].

Stress has been linked to both metabolic dysfunction and cognitive impairment, making it a potential risk factor for obesity and its associated metabolic outcomes. There is a significant neurobiological relationship between stress and the energy homeostasis system. The hypothalamic–pituitary–adrenal axis (HPA axis) is sensitive to a variety of hormones, including glucose, insulin, and ghrelin, which regulate energy balance and appetite. Additionally, the HPA axis is sensitive to the majority of peripheral and central neuropeptides, such as orexigenic neuropeptide Y, which are involved in regulating energy homeostasis. Additionally, chronic stress alters peripheral metabolism and lipid physiology, including the production of cortisol, which increases plasma levels of leukotrienes and ghrelin, and changes in neuropeptide expression that regulate energy intake. Cortisol inhibits insulin release, reduces insulin sensitivity, and causes hypermetabolism, which in turn promotes hypertension, central obesity, and glucose intolerance, all of which contribute to the development of metabolic syndrome. Finally, chronic stress prospectively predicts abdominal fat accumulation, metabolic syndrome, obesity, and other metabolic consequences. Stress can lead to irregular eating habits, which are associated with a variety of negative health effects, including increased intake of sugary and fatty foods, fast food and unhealthy snacks, bulimia, and decreased vegetable intake. Pre-existing metabolic risks may make a person more vulnerable to the negative effects of stress on body composition. For example, accumulated stress is associated with increased levels of fasting blood glucose, insulin, and insulin resistance in people with high and low BMIs, respectively. Additionally, obese people are more likely to experience increased cortisol levels. Central obesity is associated with glucocorticoid overproduction, increased basal cortisol, and increased cortisol reactivity during acute stress. These results suggest that stress promotes internal environments and behaviors that increase the risk of hypermetabolism, which can have serious long-term health consequences. Diseases such as obesity and diabetes are associated with systemic inflammation, increased oxidative stress, altered gene expression (such as telomere shortening), and decreased cognitive performance [144,145,146,147,148,149,150,151,152,153,154,155,156,157,158,159,160,161,162,163].

The impact of stress on cognitive function is noteworthy, both in the short and long term, as it leads to detrimental changes in neural structures. Acute stress can have both positive and negative effects on performance, with instances of both types of effects observed. The specific direction of these effects is influenced by various factors, including the cognitive domain investigated, proximity to cognitive processes, and the individual’s stress response. It has been observed that stress generally reduces cognitive processes that are not directly related to the stressor itself. For example, the attentional resources needed to handle stressors are prioritized. Similarly, the consolidation of information in memory is expected to be prioritized to enable future adaptive coping. Cognitive processes unrelated to an immediate threat, such as attention to one’s surroundings and searching for information not related to stress, tend to be affected. The main trigger for acute effects on cognitive function is the use of glucocorticoids, which are associated with long-term impairment of neuronal integrity and function. For example, chronically elevated plasma levels in older adults are associated with decreased hippocampal volume and hippocampal-dependent memory impairment [164,165,166,167].

**Figure 1:**
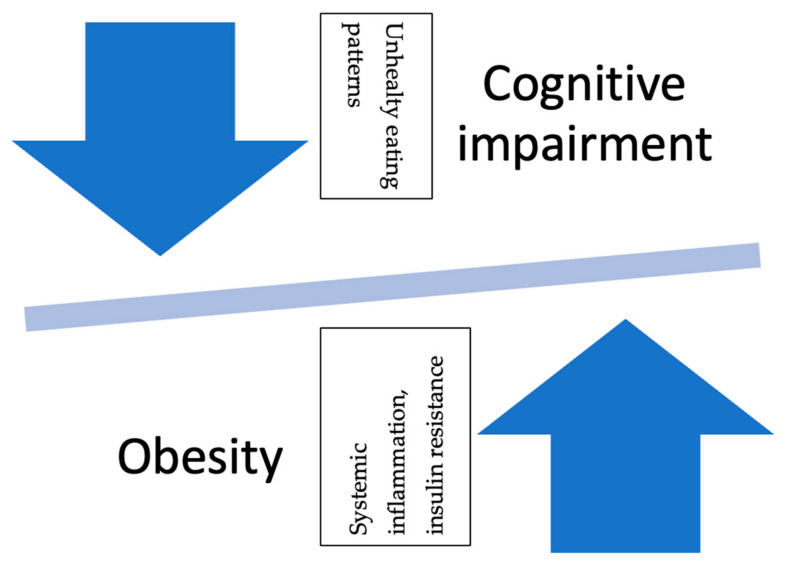
Reciprocal relationship between obesity and cognitive impairment. **Legend**: The relationship between obesity and cognitive impairment is bidirectional. Long-term obese status leads to cognitive deficits, while cognitive deficits encourage unhealthy eating patterns, which, in turn, lead to obesity.

Chronic stress leads to weight gain, especially in the central adipose tissue, through the disruption of eating behaviors and energy homeostasis. Obese individuals may be more susceptible to cognitive impairment due to higher cortisol levels during resting periods and in response to stress. This combination of increased susceptibility to stress-related effects and the metabolic dysregulation associated with obesity suggests that individuals who are obese may have a heightened risk of cognitive impairment under acute stress conditions. They examined the effects of stress-induced cognitive performance in 66 middle-aged high-weight/normal-height (HWH) adults. When exposed to a stressor, males with high WHR tended to show increased cortisol responsivity. The increased WHR was accompanied by poorer performance on declarative tasks, particularly spatial recognition memory (SGA) and paired associative learning (PAL). These results suggest that obese individuals with central adipose tissue may exhibit decreased cognitive performance under acute stress. Therefore, the increased risk of cognitive impairment observed in obese individuals may be exacerbated by increased susceptibility to harmful stressors. Additionally, there appears to be a bidirectional correlation between cognitive impairment and obesity (see Figure 1) [168].

Higher levels of the KYN metabolite kynurenic acid (KYNA) in the human brain are associated with altered fear states caused by trauma, stress, and anxiety. These elevated KYNA levels may contribute to cognitive and sensory deficits in these disorders. KYNA levels are increased in areas such as the prefrontal cortex (PFC) and cerebrospinal fluid (CSF) of patients with psychosis. Additionally, experimental manipulation of brain KYNA levels through systematic KYN administration has been shown to lead to impairment of PFC-mediated landscape shifting, spatial contextual memory, cognitive ability, fear learning, and working memory capacity. Conversely, reducing KYNA levels in the brain by pharmacological inhibition or gene deletion of KAT II, the main enzyme responsible for KYNA production in the human brain, has been shown to improve cognitive function [169].

A study by Polyak et al. has validated metabolic alterations during demyelination on both sides of the blood–brain barrier (BBB). They demonstrated the role of KYN metabolites in the animal model of demyelination generated by cuprizone (CPZ), which is comparable to progressive MS in several aspects. This study has demonstrated the profile of KYN metabolites during increasing demyelination, which also supplements the earlier evidence. Additional research could clarify the process underlying the modification of KYN metabolites and perhaps impact TRP-KYN metabolism enzyme activity [170].

## 6. Possible Reversal of Cognitive Dysfunction

In order to reduce obesity, it is recommended to lose weight through diet and exercise. There is some evidence to suggest that these interventions may also help reverse or prevent further cognitive decline. However, maintaining weight loss results requires long-term behavioral changes. Some more radical interventions, such as bariatric surgery, can result in rapid weight loss, while others, such as dieting, are slower in their outcome. Several studies have been conducted to assess cognitive function after such procedures [171].

Experimental studies have investigated the effects of specific nutrients on cognitive performance. However, understanding the impact of complete diets on cognitive function is more challenging due to the intricate nature of dietary habits and the interplay between various nutrients found in the food we consume. Research utilizing controlled whole diet approaches, which involve increasing dietary fiber intake based on Nordic Nutrition Recommendations, has demonstrated cognitive advantages for middle-aged individuals with hypertension in as little as one month. The Mediterranean dietary pattern (MDP) includes plenty of olive oil, fruits, vegetables, whole grains, legumes, and nuts, moderate intake of dairy products and alcohol, and limited intake of red meat. It is associated with a lower risk of death from the disease. Among the general public, this dietary pattern is considered healthy and recommended for people outside the Mediterranean region [172,173,174,175,176,177,178,179].

MDP has been studied in relation to cognition, cognitive decline, and dementia. Studies conducted in various settings have shown that adherence to the MDP is associated with a reduction in the number of cases. However, there are some discrepancies between the results. In a study conducted among elderly people in Spain, extra virgin olive oil (OVO) or mixed nuts were prescribed for MDP. After four years, cognitive function was compared to a control group not exposed to MDP and encouraged to reduce their dietary fat intake. Those who consumed olive oil had better verbal memory than those who did not consume olive oil. Composite memory, frontal, and global performance were preserved in both arms of the MDP compared to the group with decreased performance during tracking. Although cardiovascular risk was reduced, we could not clearly identify a single interaction mechanism between the MDP and control groups as responsible for the observed maintenance. However, different biological pathways have been proposed. These include decreased vascular risk factors, white matter pathology (including insulin resistance), metabolic disorders (including glucose metabolism, oxidative stress, and inflammation), and advanced glycation (glycation) [180,181,182,183,184,185,186,187,188,189,190,191,192,193,194].

Addressing cardiovascular risk factors in midlife through interventions may help prevent age-related cognitive decline. Studies have shown that bariatric surgery can improve memory and executive function years after surgery. However, it is important to note that people with a family history of attention deficit hyperactivity disorder (ADHD) may not experience the same cognitive benefits after bariatric surgery. This suggests that genetic predisposition or family history may impede cognitive recovery after bariatric surgery [195,196].

In people with severe obesity, bariatric surgery can cause significant weight loss, but it is unclear how it will affect cognitive performance. Li et al. conducted a meta-analysis of 20 studies gathered from PubMed, Cochrane, and Embase to estimate the impact of bariatric surgery on cognition in individuals with extreme obesity. Six of these cohort studies discovered that Roux-en-Y gastric bypass improves both short-term delayed memory function and immediate verbal memory ability. Similar effects on delayed memory function and immediate verbal memory function were found over a lengthy follow-up. When compared to the control obese group, the Roux-en-Y gastric bypass group did not demonstrate any gains in executive function, attention, or cognitive speed. Patients performed better postoperatively in all cognitive domains during repeated evaluations in 14 longitudinal investigations (12 single-arm pre-post comparative studies and 2 cohort studies in which the control group had no follow-up cognitive data). The study for the 20 operating groups revealed that, with the exception of animal fluency and letter fluency, patients who underwent bariatric surgery had greater scores than baseline scores following repeated evaluation of most neuropsychological tests. These results imply that Roux-en-Y gastric bypass may improve verbal and delayed memory function in patients with severe obesity [25,197].

## 7. Discussion

While the precise mechanisms underlying the effects of excess adipose tissue on brain function are not fully elucidated, existing research indicates that insulin resistance, inflammation, and vascular dysfunction may contribute to the observed associations between obesity and cognitive decline. This research suggests that weight loss can lead to improved brain function and cognitive outcomes, implying that obesity-related cognitive impairment may be reversible [1].

The choice of adiposity measurement used to define obesity can influence the relationship observed between obesity and dementia. The commonly used BMI does not distinguish between muscle and fat tissue, making it challenging to assess body fat accurately. Additionally, the proportion of body fat can vary with age and gender, further complicating the interpretation of BMI as a measure of obesity. Therefore, alternative methods such as waist circumference, WHR, or body composition analysis may provide a more accurate assessment of body fat distribution and its association with dementia [2].

For example, cross-sectional validation studies have shown that women have higher body fat percentages than men despite having the same BMI. Ethnicity, age, and BMI interaction were independently associated with body fat percentage in women. Using self-report data at an early age may introduce recall bias. With limited data and longer follow-up periods, it becomes difficult to draw conclusions about risk factors. These studies with longer follow-ups showed the most consistent association between obesity and dementia risk [172].

Differences in study design make it more challenging to draw conclusions about relative risks. Meta-analyses assessing the risk of adverse events associated with obesity, diabetes, and related diseases may have been affected by differences in study design, resulting in statistically nonuniform pooled. Moreover, we were unable to draw any conclusions about the effect of the timing of risk exposure. Furthermore, some studies did not adjust for ApoE status, which does not reflect its potential to modify the association between obesity and dementia risk. Other studies did not consider other relevant variables such as education, cerebrovascular injury, and stroke in their analyses. Some longitudinal studies that showed large attrition rates did not address this issue. Furthermore, the association between obesity in midlife and dementia risk in late life was more frequently reported in studies with outcome and selection biases, high attrition rates, and potentially confounding lack of adequate control of variables. Differences in diagnostic criteria for dementia and diabetes, length of follow-up, sample size, and recruitment of specific populations were found to contribute to the variation in study results. Furthermore, the issue is further complicated by the broad age range of the study sample and the inclusion of participants aged 65 and older in midlife exposure assessments. Even with the inclusion of brain imaging results and autopsy reports, we cannot determine the role of the severity of subclinical vascular disease on the risk of dementia associated with obesity. Some studies have also acknowledged cohort effects, such as a difference in the survival rate for dementia, obesity-related mortality, or perhaps being less susceptible to the negative effects of obesity, referred to as ‘the survivor effect’ [198,199,200].

The objective of one cross-sectional validation study was to examine the influence of sex, age, and race on the association between BMI and measured percentage of body fat (%fat) in a sedentary population. The analysis was conducted on 665 black and white individuals ranging in age from 17 to 65 years who were part of the Heritage Family Study cohort. After statistical analysis, the %fat of females was found to be 10.4% higher than that of males at the same BMI level. General linear model analysis indicated that age, race, and race-by-BMI interaction were independently associated with %fat in women, while in men, %fat was influenced by BMI, age, and the age-by-BMI interaction. Multiple regression analysis models elucidated these biases, and the findings underscored the nonindependence of the BMI-%fat relationship from age and gender. Moreover, they revealed a race effect among women but not men. The failure to account for these sources of bias had significant implications for defining obesity based on measured %fat [198,201,202,203].

Retrospective studies, despite their valuable role in medical research, do present a set of challenges primarily stemming from their observational nature. One of the foremost difficulties lies in establishing causation and relying on existing data, often collected for different purposes, making it challenging to control for confounding variables or establish a clear cause-and-effect relationship. Also, we battled with selection, recall, and information bias, as the accuracy and completeness of historical data can be compromised. While these types of studies can provide insights into the association between variables, caution is necessary when interpreting results and making definitive causal claims in the absence of experimental control. Our research is valuable in synthesizing existing literature but also has limitations worth considering. It may be subject to publication bias, as studies with positive findings are more likely to be published, potentially skewing the overall conclusions. The causation and the direction of the relationship between obesity and cognitive function can remain ambiguous. The method and means of measuring the effect of obesity on cognition are different in many studies and can be considered a limitation, as the problematic assessment of body fat, the self-reported data, the follow-up, the period of time the person was obese, the different protocol used earlier in diagnosing obesity, and so on. Moreover, the generalizability of findings may be limited, as obesity’s impact on cognition can vary depending on many factors, such as age, gender, social status, and comorbidities, which are not fully accounted for in the reviewed studies [204,205,206,207].

The research conducted worldwide concerning obesity-induced cognitive impairment has significant practical implications for various stakeholders, including healthcare providers, policymakers, clinical practices, and individuals [208,209,210,211].

As far as clinical interventions go, research in this field informs healthcare providers about the cognitive consequences of obesity, highlighting the need for comprehensive assessments and tailored interventions. Practitioners can now integrate cognitive assessments into their clinical evaluations and provide guidance on weight management strategies that may improve cognitive outcomes or maybe refer these patients to the selected specialists [208,209,210,211].

In the sector of public health, policymakers can use findings from the medical core to develop public health policies aimed at obesity prevention and management, which might involve implementing programs promoting healthier lifestyles, encouraging physical activity, and regulating the availability of high-calorie, low-nutrient foods [208,209,210,211].

For patient education, individuals struggling with obesity can benefit from a better understanding of the cognitive risks associated with excess weight, and maybe knowledge of a potential cognitive impairment can serve as a motivating factor for weight management and a healthier lifestyle [208,209,210].

The emerging understanding that weight loss can enhance brain function and cognitive outcomes offers promising clinical implications and opens new avenues for future research. From a clinical perspective, these findings underscore the significance of weight management and lifestyle interventions in addressing not only the physical health consequences of obesity but also the potential reversibility of obesity-related cognitive impairment and cognitive well-being. Healthcare professionals can integrate cognitive assessments into weight management programs to monitor changes in cognitive function as patients lose weight. Furthermore, these insights suggest that targeting weight loss as a therapeutic approach for cognitive decline, potentially even in conditions like obesity-related cognitive impairment or mild cognitive impairment, holds promise. Future research could delve deeper into the mechanisms underpinning the reversal of cognitive impairment through weight loss, exploring factors such as improved cardiovascular health, reduced inflammation, and hormonal changes. The role of various interventions, such as dietary modifications, exercise regimens, and bariatric surgery, could prove helpful in optimizing cognitive outcomes. Additionally, exploring the long-term sustainability and durability of cognitive improvements following weight loss and the potential interplay with genetic, metabolic, and neurobiological factors can be a rich area for further investigation. Ultimately, the potential reversibility of obesity-related cognitive impairment through weight loss highlights a novel and encouraging dimension of holistic healthcare and warrants further exploration to refine clinical interventions and therapeutic strategies [211,212].

Future research on the relationship between obesity and cognition should continue to explore the intricate mechanisms that link these two factors and their potential implications for public health and clinical practice. Investigating the neurobiological underpinnings of cognitive impairments in obese individuals, such as the impact of chronic inflammation, insulin resistance, and altered adipokine levels on brain structure and function, remains an essential subject for further exploration. Moreover, longitudinal studies that gather a wider range of age groups and diverse populations could shed more light on how obesity affects cognition over time and whether there is a critical window of susceptibility in which we can intervene with medication, exercise, lifestyle modifications, or bariatric surgery. Research on the potential cognitive benefits of weight loss and the role of individualized strategies in cognitive impairment is also a promising direction for future studies in this field [213,214].

It has been demonstrated that the essential oils of *Chrysanthemum morifolium* Ramat., *Zingiber officinale* Roscoe, *Santalum album* L., *Citrus reticulata* Blanco, *Atractylodes lancea*, and *Myristica fragrans* Houtt. have neuroprotective or anti-neuroinflammatory properties in vitro and provide fast-acting antidepressant effects when administered intranasally to mice. Notably, through interactions with NMDARs and AMPARs, essential oils from *Chrysanthemum morifolium* Ramat. and *Atractylodes lancea*, as well as their volatile compounds, such as atractylon, α-curcumene, α farnesene, and selina-4(14),7(11)-dien-8-one, may be useful candidates for development as rapid-acting intranasal antidepressants. Validating the safety of these essential oils and chemicals for intranasal delivery, as well as the precise mechanism underlying their fast-acting antidepressant benefits, are critical for future research. They should also be investigated with a particular animal disease model [215].

Despite extensive research in this field, several critical questions remain unanswered, leaving room for further investigations. After unraveling the intricate mechanisms that connect obesity to cognitive decline, we believe that the impact of obesity at different life stages and its interaction with genetic, environmental, and lifestyle factors on cognition outcomes require further investigations. Also, more long-term studies, including clinical trials, are needed to evaluate the effectiveness of various interventions in preventing and mitigating cognitive decline in obese individuals, so reversibility of cognitive impairment in this population is another crucial question that remains on the table. More research is needed to understand the threshold at which obesity becomes a significant risk factor for cognitive decline and the nuances of this relationship across more types of populations in order to answer this critical public health issue [213,214].

## 8. Conclusions

The implications of this research can lead to the development of interventions that may include cognitive rehabilitation programs, lifestyle modifications, and pharmacological interventions. Employers and educational institutions can consider the cognitive impact of obesity when developing workplace wellness programs or support services for students struggling with weight issues. In summary, research on obesity-induced cognitive impairment and the effect that obesity has on our brain has practical implications that extend to healthcare, public policies, education, and society at large, contributing to reducing the social stigma and discrimination against individuals with obesity. It amplifies the importance of addressing both the physical and cognitive aspects of obesity to improve overall health and well-being. 

Most research highlights the impact of vascular dysfunction and systemic inflammation on the nervous system in patients with obesity and the subsequent neurological changes. Obesity during the early to mid-ages leads to an earlier onset of cognitive dysfunction in various forms. Also, lifestyle intervention can reverse cognitive dysfunction, especially dieting, to encourage weight loss.

## Figures and Tables

**Table 1:** MCI Working Group of the European Consortium on Alzheimer’s Disease, Brescia Meeting, Italy, June 2005 Criteria.

Criteria	References
Cognitive impairment reported by patients or their families	[96,97]
Reported decline in cognitive functioning relative to previous abilities during the past year by the patient or informant	
Cognitive disorders evidenced during clinical evaluation	
Absence of major repercussions on the daily life of the patient	
Absent dementia	

**Legend**: These are the criteria currently in use and recommended as standard for establishing the diagnosis of mild cognitive impairment.

## Data Availability

Not applicable.

## References

[1] Tanaka H., Gourley D.D., Dekhtyar M., Haley A.P. (2020). Cognition, Brain Structure, and Brain Function in Individuals with Obesity and Related Disorders. Curr. Obes. Rep..

[2] Ross R., Neeland I.J., Yamashita S., Shai I., Seidell J., Magni P., Santos R.D., Arsenault B., Cuevas A., Hu F.B. (2020). Waist circumference as a vital sign in clinical practice: A Consensus Statement from the IAS and ICCR Working Group on Visceral Obesity. Nat. Rev. Endocrinol..

[3] Alberti K.G., Eckel R.H., Grundy S.M., Zimmet P.Z., Cleeman J.I., Donato K.A., Fruchart J.C., James W.P., Loria C.M., Smith S.C. (2009). Harmonizing the metabolic syndrome: A joint interim statement of the International Diabetes Federation Task Force on Epidemiology and Prevention; National Heart, Lung, and Blood Institute; American Heart Association; World Heart Federation; International Atherosclerosis Society; and International Association for the Study of Obesity. Circulation.

[4] Janssen F., Bardoutsos A., El Gewily S., De Beer J. (2021). Future life expectancy in Europe taking into account the impact of smoking, obesity, and alcohol. eLife.

[5] Yankner B.A., Lu T., Loerch P. (2008). The aging brain. Annu. Rev. Pathol..

[6] Alzheimer’s Disease International (2015). World Alzheimer Report 2015. The Global Impact of Dementia: An Analysis of Prevalence, Incidence, Cost and Trends.

[7] Mortimer J.A., Borenstein A.R., Gosche K.M., Snowdon D.A. (2005). Very early detection of Alzheimer neuropathology and the role of brain reserve in modifying its clinical expression. J. Geriatr. Psychiatry Neurol..

[8] Amieva H., Jacqmin-Gadda H., Orgogozo J.M., Le Carret N., Helmer C., Letenneur L., Barberger-Gateau P., Fabrigoule C., Dartigues J.F. (2005). The 9 year cognitive decline before dementia of the Alzheimer type: A prospective population-based study. Brain.

[9] Karch C.M., Cruchaga C., Goate A.M. (2014). Alzheimer’s disease genetics: From the bench to the clinic. Neuron.

[10] De Oliveira F.F., de Almeida S.S., Chen E.S., Smith M.C., Naffah-Mazzacoratti M.D.G., Bertolucci P.H.F. (2018). Lifetime Risk Factors for Functional and Cognitive Outcomes in Patients with Alzheimer’s Disease. J. Alzheimers Dis..

[11] De la Torre J.C. (2010). Alzheimer’s disease is incurable but preventable. J. Alzheimers Dis..

[12] Coley N., Andrieu S., Gardette V., Gillette-Guyonnet S., Sanz C., Vellas B., Grand A. (2008). Dementia prevention: Methodological explanations for inconsistent results. Epidemiol. Rev..

[13] Middleton L.E., Yaffe K. (2009). Promising strategies for the prevention of dementia. Arch. Neurol..

[14] Singh-Manoux A., Kivimaki M., Glymour M.M., Elbaz A., Berr C., Ebmeier K.P., Ferrie J.E., Dugravot A. (2012). Timing of onset of cognitive decline: Results from Whitehall II prospective cohort study. BMJ.

[15] WHO Europe (2014). European Food and Nutrition Action Plan 2015–2020. http://www.euro.who.int/__data/assets/pdf_file/0008/253727/64wd14e_FoodNutAP_140426.pdf.

[16] Gunstad J., Lhotsky A., Wendell C.R., Ferrucci L., Zonderman A.B. (2010). Longitudinal examination of obesity and cognitive function: Results from the Baltimore longitudinal study of aging. Neuroepidemiology.

[17] Lamport D.J., Dye L., Mansfield M.W., Lawton C.L. (2013). Acute glycaemic load breakfast manipulations do not attenuate cognitive impairments in adults with type 2 diabetes. Clin. Nutr..

[18] Lamport D.J., Lawton C.L., Mansfield M.W., Moulin C.A., Dye L. (2014). Type 2 diabetes and impaired glucose tolerance are associated with word memory source monitoring recollection deficits but not simple recognition familiarity deficits following water, low glycaemic load, and high glycaemic load breakfasts. Physiol. Behav..

[19] Yaffe K., Kanaya A., Lindquist K., Simonsick E.M., Harris T., Shorr R.I., Tylavsky F.A., Newman A.B. (2004). The metabolic syndrome, inflammation, and risk of cognitive decline. JAMA.

[20] Gong H.J., Tang X., Chai Y.H., Qiao Y.S., Xu H., Patel I., Zhang J.Y., Simó R., Zhou J.B. (2023). Relationship Between Weight-Change Patterns and Cognitive Function: A Retrospective Study. J. Alzheimers Dis..

[21] Chuang S.Y., Liu W.L., Chang H.Y., Hsu C.C., Pan W.H. (2023). Central obesity and elevated blood pressure in middle life are associated with physical and cognitive impairment in later life: A retrospective design with repeated measures. Exp. Gerontol..

[22] Twig G., Tirosh A., Derazne E., Haklai Z., Goldberger N., Afek A., Gerstein H.C., Kark J.D., Cukierman-Yaffe T. (2018). Cognitive function in adolescence and the risk for premature diabetes and cardiovascular mortality in adulthood. Cardiovasc. Diabetol..

[23] Vanhandsaeme G., Benhalima K. (2021). The long-term metabolic and neurocognitive risks in offspring of women with type 1 diabetes mellitus. Acta Diabetol..

[24] Cukierman-Yaffe T., Kasher-Meron M., Fruchter E., Gerstein H.C., Afek A., Derazne E., Tzur D., Karasik A., Twig G. (2015). Cognitive Performance at Late Adolescence and the Risk for Impaired Fasting Glucose Among Young Adults. J. Clin. Endocrinol. Metab..

[25] Custers E., Vreeken D., Kaufmann L.K., Pujol-Gualdo N., Asbreuk M., Wiesmann M., Aarts E., Hazebroek E.J., Kiliaan A.J. (2023). Cognitive Control and Weight Loss After Bariatric Surgery: The BARICO Study. Obes. Surg..

[26] Boidin M., Handfield N., Ribeiro P.A.B., Desjardins-Crépeau L., Gagnon C., Lapierre G., Gremeaux V., Lalongé J., Nigam A., Juneau M. (2020). Obese but Fit: The Benefits of Fitness on Cognition in Obese Older Adults. Can. J. Cardiol..

[27] Lampe L., Zhang R., Beyer F., Huhn S., Kharabian Masouleh S., Preusser S., Bazin P.L., Schroeter M.L., Villringer A., Witte A.V. (2019). Visceral obesity relates to deep white matter hyperintensities via inflammation. Ann. Neurol..

[28] Papageorgiou I., Astrakas L.G., Xydis V., Alexiou G.A., Bargiotas P., Tzarouchi L., Zikou A.K., Kiortsis D.N., Argyropoulou M.I. (2017). Abnormalities of brain neural circuits related to obesity: A Diffusion Tensor Imaging study. Magn. Reson. Imaging.

[29] Marqués-Iturria I., Pueyo R., Garolera M., Segura B., Junqué C., García-García I., José Sender-Palacios M., Vernet-Vernet M., Narberhaus A., Ariza M. (2013). Frontal cortical thinning and subcortical volume reductions in early adulthood obesity. Psychiatry Res..

[30] Iceta S., Dadar M., Daoust J., Scovronec A., Leblanc V., Pelletier M., Biertho L., Tchernof A., Bégin C., Michaud A. (2021). Association between Visceral Adiposity Index, Binge Eating Behavior, and Grey Matter Density in Caudal Anterior Cingulate Cortex in Severe Obesity. Brain Sci..

[31] Pflanz C.P., Tozer D.J., Harshfield E.L., Tay J., Farooqi S., Markus H.S. (2022). Central obesity is selectively associated with cerebral gray matter atrophy in 15,634 subjects in the UK Biobank. Int. J. Obes..

[32] Morys F., Potvin O., Zeighami Y., Vogel J., Lamontagne-Caron R., Duchesne S., Dagher A. (2023). Obesity-Associated Neurodegeneration Pattern Mimics Alzheimer’s Disease in an Observational Cohort Study. J. Alzheimers Dis..

[33] Dake M.D., De Marco M., Blackburn D.J., Wilkinson I.D., Remes A., Liu Y., Pikkarainen M., Hallikainen M., Soininen H., Venneri A. (2021). Obesity and Brain Vulnerability in Normal and Abnormal Aging: A Multimodal MRI Study. J. Alzheimers Dis. Rep..

[34] Bourassa K., Sbarra D.A. (2017). Body mass and cognitive decline are indirectly associated via inflammation among aging adults. Brain Behav. Immun..

[35] Balasubramanian P., Kiss T., Tarantini S., Nyúl-Tóth Á., Ahire C., Yabluchanskiy A., Csipo T., Lipecz A., Tabak A., Institoris A. (2021). Obesity-induced cognitive impairment in older adults: A microvascular perspective. Am. J. Physiol. Heart Circ. Physiol..

[36] Buie J.J., Watson L.S., Smith C.J., Sims-Robinson C. (2019). Obesity-related cognitive impairment: The role of endothelial dysfunction. Neurobiol. Dis..

[37] De Paula G.C., Brunetta H.S., Engel D.F., Gaspar J.M., Velloso L.A., Engblom D., de Oliveira J., de Bem A.F. (2021). Hippocampal Function Is Impaired by a Short-Term High-Fat Diet in Mice: Increased Blood-Brain Barrier Permeability and Neuroinflammation as Triggering Events. Front. Neurosci..

[38] De Vargas L.D.S., Jantsch J., Fontoura J.R., Dorneles G.P., Peres A., Guedes R.P. (2023). Effects of Zinc Supplementation on Inflammatory and Cognitive Parameters in Middle-Aged Women with Overweight or Obesity. Nutrients.

[39] Wang J., Li L., Zhang Z., Zhang X., Zhu Y., Zhang C., Bi Y. (2022). Extracellular vesicles mediate the communication of adipose tissue with brain and promote cognitive impairment associated with insulin resistance. Cell Metab..

[40] Reilly S., Saltiel A. (2017). Adapting to obesity with adipose tissue inflammation. Nat. Rev. Endocrinol..

[41] Rhea E.M., Salameh T.S., Logsdon A.F., Hanson A.J., Erickson M.A., Banks W.A. (2017). Blood-Brain Barriers in Obesity. AAPS J..

[42] Andrade M.M., Fernandes C., Forny-Germano L., Gonçalves R.A., Gomes M., Castro-Fonseca E., Ramos-Lobo A.M., Tovar-Moll F., Andrade-Moraes C.H., Donato J. (2023). Alteration in the number of neuronal and non-neuronal cells in mouse models of obesity. Brain Commun..

[43] Baufeld C., Osterloh A., Prokop S., Miller K.R., Heppner F.L. (2016). High-fat diet-induced brain region-specific phenotypic spectrum of CNS resident microglia. Acta Neuropathol..

[44] Kamitakahara A., Bouyer K., Wang C.H., Simerly R. (2018). A critical period for the trophic actions of leptin on AgRP neurons in the arcuate nucleus of the hypothalamus. J. Comp. Neurol..

[45] Izquierdo A.G., Crujeiras A.B., Casanueva F.F., Carreira M.C. (2019). Leptin, Obesity, and Leptin Resistance: Where Are We 25 Years Later. Nutrients.

[46] Irving A., Harvey J. (2021). Regulation of hippocampal synaptic function by the metabolic hormone leptin: Implications for health and disease. Prog. Lipid Res..

[47] Mooldijk S.S., Ikram M.K., Ikram M.A. (2022). Adiponectin, Leptin, and Resistin and the Risk of Dementia. J. Gerontol. A Biol. Sci. Med. Sci..

[48] Bednarska-Makaruk M., Graban A., Wiśniewska A., Łojkowska W., Bochyńska A., Gugała-Iwaniuk M., Sławińska K., Ługowska A., Ryglewicz D., Wehr H. (2017). Association of adiponectin, leptin and resistin with inflammatory markers and obesity in dementia. Biogerontology.

[49] Fan X., Yuan W., Huang W., Lin Z. (2023). Recent progress in leptin signaling from a structural perspective and its implications for diseases. Biochimie.

[50] Zhang X., Zhang G., Zhang H., Karin M., Bai H., Cai D. (2008). Hypothalamic IKKbeta/NF-kappaB and ER stress link overnutrition to energy imbalance and obesity. Cell.

[51] Segura S., Efthimiadi L., Porcher C., Courtes S., Coronas V., Krantic S., Moyse E. (2015). Leptin-dependent neurotoxicity via induction of apoptosis in adult rat neurogenic cells. Front. Cell Neurosci..

[52] Lassenius M.I., Pietiläinen K.H., Kaartinen K., Pussinen P.J., Syrjänen J., Forsblom C., Pörsti I., Rissanen A., Kaprio J., Mustonen J. (2011). Bacterial endotoxin activity in human serum is associated with dyslipidemia, insulin resistance, obesity, and chronic inflammation. Diabetes Care.

[53] Lam Y.Y., Ha C.W., Campbell C.R., Mitchell A.J., Dinudom A., Oscarsson J., Cook D.I., Hunt N.H., Caterson I.D., Holmes A.J. (2012). Increased gut permeability and microbiota change associate with mesenteric fat inflammation and metabolic dysfunction in diet-induced obese mice. PLoS ONE.

[54] Inaba T., Yamashiro K., Kurita N., Ueno Y., Miyamoto N., Hira K., Nakajima S., Kijima C., Nakaguro R., Urabe T. (2023). Microbial lipopolysaccharide-induced inflammation contributes to cognitive impairment and white matter lesion progression in diet-induced obese mice with chronic cerebral hypoperfusion. CNS Neurosci. Ther..

[55] Dong T.S., Guan M., Mayer E.A., Stains J., Liu C., Vora P., Jacobs J.P., Lagishetty V., Chang L., Barry R.L. (2022). Obesity is associated with a distinct brain-gut microbiome signature that connects Prevotella and Bacteroides to the brain’s reward center. Gut Microbes..

[56] Arnoriaga-Rodríguez M., Mayneris-Perxachs J., Burokas A., Contreras-Rodríguez O., Blasco G., Coll C., Biarnés C., Miranda-Olivos R., Latorre J., Moreno-Navarrete J.M. (2020). Obesity Impairs Short-Term and Working Memory through Gut Microbial Metabolism of Aromatic Amino Acids. Cell Metab..

[57] Ge X., Zheng M., Hu M., Fang X., Geng D., Liu S., Wang L., Zhang J., Guan L., Zheng P. (2023). Butyrate ameliorates quinolinic acid-induced cognitive decline in obesity models. J. Clin. Investig..

[58] Favennec M., Hennart B., Caiazzo R., Leloire A., Yengo L., Verbanck M., Arredouani A., Marre M., Pigeyre M., Bessede A. (2015). The kynurenine pathway is activated in human obesity and shifted toward kynurenine monooxygenase activation. Obesity.

[59] Parada Venegas D., De la Fuente M.K., Landskron G., González M.J., Quera R., Dijkstra G., Harmsen H.J.M., Faber K.N., Hermoso M.A. (2019). Short Chain Fatty Acids (SCFAs)-Mediated Gut Epithelial and Immune Regulation and Its Relevance for Inflammatory Bowel Diseases. Front. Immunol..

[60] Sun Y., Zhang H., Zhang X., Wang W., Chen Y., Cai Z., Wang Q., Wang J., Shi Y. (2023). Promotion of astrocyte-neuron glutamate-glutamine shuttle by SCFA contributes to the alleviation of Alzheimer’s disease. Redox. Biol..

[61] Colombo A.V., Sadler R.K., Llovera G., Singh V., Roth S., Heindl S., Sebastian Monasor L., Verhoeven A., Peters F., Parhizkar S. (2021). Microbiota-derived short chain fatty acids modulate microglia and promote Aβ plaque deposition. eLife.

[62] Kim K.N., Yao Y., Ju S.Y. (2019). Short Chain Fatty Acids and Fecal Microbiota Abundance in Humans with Obesity: A Systematic Review and Meta-Analysis. Nutrients.

[63] Suarez A.N., Hsu T.M., Liu C.M., Noble E.E., Cortella A.M., Nakamoto E.M., Hahn J.D., de Lartigue G., Kanoski S.E. (2018). Gut vagal sensory signaling regulates hippocampus function through multi-order pathways. Nat. Commun..

[64] Harvey P.D. (2019). Domains of cognition and their assessment. Dialogues Clin. Neurosci..

[65] Gierach M., Rasmus A., Orłowska E. (2022). Verbal Fluency in Metabolic Syndrome. Brain Sci..

[66] Koutsonida M., Koskeridis F., Markozannes G., Kanellopoulou A., Mousas A., Ntotsikas E., Ioannidis P., Aretouli E., Tsilidis K.K. (2023). Metabolic syndrome and cognitive deficits in the Greek cohort of Epirus Health Study. Neurol. Sci..

[67] Heriseanu A.I., Hay P., Touyz S. (2021). A cross-sectional examination of executive function and its associations with grazing in persons with obesity with and without eating disorder features compared to a healthy control group. Eat. Weight Disord..

[68] Fernandes A.C., Viegas Â.A., Lacerda A.C.R., Nobre J.N.P., Morais R.L.S., Figueiredo P.H.S., Costa H.S., Camargos A.C.R., Ferreira F.O., de Freitas P.M. (2022). Association between executive functions and gross motor skills in overweight/obese and eutrophic preschoolers: Cross-sectional study. BMC Pediatr..

[69] Banjevic B., Aleksic D., Aleksic Veljkovic A., Katanic B., Masanovic B. (2022). Differences between Healthy-Weight and Overweight Serbian Preschool Children in Motor and Cognitive Abilities. Int. J. Env. Res. Public Health.

[70] Ma F.F., Luo D.M. (2023). Relationships between physical activity, fundamental motor skills, and body mass index in preschool children. Front. Public Health.

[71] Salama I.I., Abdelrahman A.H., Salama S.I., Abdellatif G.A., Rabah T.M., Saleh R.M., Elmosalami D.M., Rabah A.M., Fouad W.A. (2018). Obesity and Predictors Affecting the Occurrence of Mild Cognitive Impairment. Res. J. Pharm. Biol. Chem. Sci..

[72] Rodrigue C., Ouellette A.S., Lemieux S., Tchernof A., Biertho L., Bégin C. (2018). Executive functioning and psychological symptoms in food addiction: A study among individuals with severe obesity. Eat Weight Disord..

[73] Pietrabissa G., Cammisuli D.M., Scarpina F., Volpi C., Crotti L., Mauro A., Gondoni L.A., Castelnuovo G. (2023). Executive Attentional Dyscontrol as a Core Cognitive and Behavioral Feature of Individuals with Obesity and Cardiovascular Disease: A Cross-Sectional Investigation. Brain Sci..

[74] Perpiñá C., Segura M., Sánchez-Reales S. (2017). Cognitive flexibility and decision-making in eating disorders and obesity. Eat Weight Disord..

[75] Yang Y., Shields G.S., Guo C., Liu Y. (2018). Executive function performance in obesity and overweight individuals: A meta-analysis and review. Neurosci. Biobehav. Rev..

[76] Gilewski M.J., Zelinski E.M., Schaie K.W. (1990). The memory functioning questionnaire for assessment of memory complaints in adulthood and old age. Psychol. Aging.

[77] Rukadikar C., Shah C.J., Raju A., Popat S., Josekutty R. (2023). The Influence of Obesity on Cognitive Functioning Among Healthcare Professionals: A Comprehensive Analysis. Cureus.

[78] Alcaraz-Ortíz M.R., Ramírez-Flores D., Palafox-López G.I., Reyes-Hernández J.U. (2015). El déficit cognitivo relacionado con el índice de masa corporal elevado. Rev. Esp. Cienc. Salud..

[79] Alarcón G., Ray S., Nagel B.J. (2016). Lower Working Memory Performance in Overweight and Obese Adolescents Is Mediated by White Matter Microstructure. J. Int. Neuropsychol. Soc..

[80] De Wit L., Kirton J.W., O’Shea D.M., Szymkowicz S.M., McLaren M.E., Dotson V.M. (2017). Effects of body mass index and education on verbal and nonverbal memory. Neuropsychol. Dev. Cogn. B Aging Neuropsychol. Cogn..

[81] Cheke L.G., Simons J.S., Clayton N.S. (2016). Higher body mass index is associated with episodic memory deficits in young adults. Q. J. Exp. Psychol..

[82] Eichen D.M., Kang Sim D.E., Appleton-Knapp S.L., Strong D.R., Boutelle K.N. (2023). Adults with overweight or obesity use less efficient memory strategies compared to adults with healthy weight on a verbal list learning task modified with food words. Appetite.

[83] Flegal K.M., Kit B.K., Orpana H., Graubard B.I. (2013). Association of all-cause mortality with overweight and obesity using standard body mass index categories: A systematic review and meta-analysis. JAMA.

[84] Xu W.L., Atti A.R., Gatz M., Pedersen N.L., Johansson B., Fratiglioni L. (2011). Midlife overweight and obesity increase late-life dementia risk: A population-based twin study. Neurology.

[85] Whitmer R.A., Gunderson E.P., Barrett-Connor E., Quesenberry C.P., Yaffe K. (2005). Obesity in middle age and future risk of dementia: A 27 year longitudinal population based study. BMJ.

[86] Cournot M., Marquié J.C., Ansiau D., Martinaud C., Fonds H., Ferrières J., Ruidavets J.B. (2006). Relation between body mass index and cognitive function in healthy middle-aged men and women. Neurology.

[87] Gunstad J., Paul R.H., Cohen R.A., Tate D.F., Gordon E. (2006). Obesity is associated with memory deficits in young and middle-aged adults. Eat. Weight Disord..

[88] Cheke L.G., Bonnici H.M., Clayton N.S., Simons J.S. (2017). Obesity and insulin resistance are associated with reduced activity in core memory regions of the brain. Neuropsychologia.

[89] Coppin G., Nolan-Poupart S., Jones-Gotman M., Small D.M. (2014). Working memory and reward association learning impairments in obesity. Neuropsychologia.

[90] Conforto R.M., Gershman L. (1985). Cognitive processing differences between obese and nonobese subjects. Addict. Behav..

[91] Fergenbaum J.H., Bruce S., Lou W., Hanley A.J., Greenwood C., Young T.K. (2009). Obesity and lowered cognitive performance in a Canadian First Nations population. Obesity.

[92] Boeka A.G., Lokken K.L. (2008). Neuropsychological performance of a clinical sample of extremely obese individuals. Arch. Clin. Neuropsychol..

[93] Gonzales M.M., Tarumi T., Miles S.C., Tanaka H., Shah F., Haley A.P. (2010). Insulin sensitivity as a mediator of the relationship between BMI and working memory-related brain activation. Obesity.

[94] Fagundo A.B., de la Torre R., Jiménez-Murcia S., Agüera Z., Granero R., Tárrega S., Botella C., Baños R., Fernández-Real J.M., Rodríguez R. (2012). Executive functions profile in extreme eating/weight conditions: From anorexia nervosa to obesity. PLoS ONE.

[95] Lokken K.L., Boeka A.G., Yellumahanthi K., Wesley M., Clements R.H. (2010). Cognitive performance of morbidly obese patients seeking bariatric surgery. Am. Surg..

[96] Portet F., Ousset P.J., Visser P.J., Frisoni G.B., Nobili F., Scheltens P., Vellas B., Touchon J. (2006). MCI Working Group of the European Consortium on Alzheimer’s Disease (EADC). Mild cognitive impairment (MCI) in medical practice: A critical review of the concept and new diagnostic procedure. Report of the MCI Working Group of the European Consortium on Alzheimer’s Disease. J. Neurol. Neurosurg. Psychiatry.

[97] Oprescu A.C., Grosu C., Bild W. (2023). Correlations between Cognitive Evaluation and Metabolic Syndrome. Metabolites.

[98] Cavedini P., Bassi T., Ubbiali A., Casolari A., Giordani S., Zorzi C., Bellodi L. (2004). Neuropsychological investigation of decision-making in anorexia nervosa. Psychiatry Res..

[99] Boeka A.G., Lokken K.L. (2006). The Iowa gambling task as a measure of decision making in women with bulimia nervosa. J. Int. Neuropsychol. Soc..

[100] Brogan A., Hevey D., O’Callaghan G., Yoder R., O’Shea D. (2011). Impaired decision making among morbidly obese adults. J. Psychosom. Res..

[101] Davis C., Patte K., Curtis C., Reid C. (2010). Immediate pleasures and future consequences. A neuropsychological study of binge eating and obesity. Appetite.

[102] Fitzpatrick S., Gilbert S., Serpell L. (2013). Systematic review: Are overweight and obese individuals impaired on behavioural tasks of executive functioning. Neuropsychol. Rev..

[103] Prickett C., Brennan L., Stolwyk R. (2015). Examining the relationship between obesity and cognitive function: A systematic literature review. Obes. Res. Clin. Pract..

[104] Miller A.A., Spencer S.J. (2014). Obesity and neuroinflammation: A pathway to cognitive impairment. Brain. Behav. Immun..

[105] Ward M.A., Carlsson C.M., Trivedi M.A., Sager M.A., Johnson S.C. (2005). The effect of body mass index on global brain volume in middle-aged adults: A cross sectional study. BMC Neurol..

[106] Gunstad J., Paul R.H., Cohen R.A., Tate D.F., Spitznagel M.B., Grieve S., Gordon E. (2008). Relationship between body mass index and brain volume in healthy adults. Int. J. Neurosci..

[107] Shefer G., Marcus Y., Stern N. (2013). Is obesity a brain disease. Neurosci. Biobehav. Rev..

[108] Verstynen T.D., Weinstein A.M., Schneider W.W., Jakicic J.M., Rofey D.L., Erickson K.I. (2012). Increased body mass index is associated with a global and distributed decrease in white matter microstructural integrity. Psychosom. Med..

[109] Willeumier K.C., Taylor D.V., Amen D.G. (2011). Elevated BMI is associated with decreased blood flow in the prefrontal cortex using SPECT imaging in healthy adults. Obesity.

[110] Battaglia S., Schmidt A., Hassel S., Tanaka M. (2023). Editorial: Case reports in neuroimaging and stimulation. Front. Psychiatry.

[111] Ronan L., Alexander-Bloch A.F., Wagstyl K., Farooqi S., Brayne C., Tyler L.K., Fletcher P.C. (2016). Obesity associated with increased brain age from midlife. Neurobiol. Aging.

[112] Fotenos A.F., Snyder A.Z., Girton L.E., Morris J.C., Buckner R.L. (2005). Normative estimates of cross-sectional and longitudinal brain volume decline in aging and AD. Neurology.

[113] Kershaw E.E., Flier J.S. (2004). Adipose tissue as an endocrine organ. J. Clin. Endocrinol. Metab..

[114] Makki K., Froguel P., Wolowczuk I. (2013). Adipose tissue in obesity-related inflammation and insulin resistance: Cells, cytokines, and chemokines. ISRN Inflamm..

[115] Kaser S., Tatarczyk T., Stadlmayr A., Ciardi C., Ress C., Tschoner A., Sandhofer A., Paulweber B., Ebenbichler C.F., Patsch J.R. (2008). Effect of obesity and insulin sensitivity on adiponectin isoform distribution. Eur. J. Clin. Investig..

[116] Nigro E., Scudiero O., Monaco M.L., Palmieri A., Mazzarella G., Costagliola C., Bianco A., Daniele A. (2014). New insight into adiponectin role in obesity and obesity-related diseases. Biomed. Res. Int..

[117] Bolzenius J.D., Laidlaw D.H., Cabeen R.P., Conturo T.E., McMichael A.R., Lane E.M., Heaps J.M., Salminen L.E., Baker L.M., Gunstad J. (2013). Impact of body mass index on neuronal fiber bundle lengths among healthy older adults. Brain Imaging Behav..

[118] Beydoun M.A., Beydoun H.A., Wang Y. (2008). Obesity and central obesity as risk factors for incident dementia and its subtypes: A systematic review and meta-analysis. Obes. Rev..

[119] Whitmer R.A., Gustafson D.R., Barrett-Connor E., Haan M.N., Gunderson E.P., Yaffe K. (2008). Central obesity and increased risk of dementia more than three decades later. Neurology.

[120] Baumgart M., Snyder H.M., Carrillo M.C., Fazio S., Kim H., Johns H. (2015). Summary of the evidence on modifiable risk factors for cognitive decline and dementia: A population-based perspective. Alzheimers Dement..

[121] Kim S., Kim Y., Park S.M. (2016). Body Mass Index and Decline of Cognitive Function. PLoS ONE.

[122] Hughes T.F., Borenstein A.R., Schofield E., Wu Y., Larson E.B. (2009). Association between late-life body mass index and dementia: The Kame Project. Neurology.

[123] Wotton C.J., Goldacre M.J. (2014). Age at obesity and association with subsequent dementia: Record linkage study. Postgrad. Med. J..

[124] Keage H.A., Gupta S., Brayne C., Aarsland D., Brayne C., Gupta S., Keage H.A., Seshasai S.R., Llewellyn D., McDougall F. (2011). Risk for dementia and age at measurement. Int. J. Geriatr. Psychiatry.

[125] Kloppenborg R.P., van den Berg E., Kappelle L.J., Biessels G.J. (2008). Diabetes and other vascular risk factors for dementia: Which factor matters most? A systematic review. Eur. J. Pharmacol..

[126] Albanese E., Launer L.J., Egger M., Prince M.J., Giannakopoulos P., Wolters F.J., Egan K. (2017). Body mass index in midlife and dementia: Systematic review and meta-regression analysis of 589,649 men and women followed in longitudinal studies. Alzheimers Dement..

[127] Luchsinger J.A., Patel B., Tang M.X., Schupf N., Mayeux R. (2007). Measures of adiposity and dementia risk in elderly persons. Arch Neurol..

[128] Luchsinger J.A., Gustafson D.R. (2009). Adiposity and Alzheimer’s disease. Curr. Opin. Clin. Nutr. Metab. Care.

[129] Stevens J., McClain J.E., Truesdale K.P. (2008). Selection of measures in epidemiologic studies of the consequences of obesity. Int. J. Obesity.

[130] Caruana E.J., Roman M., Hernández-Sánchez J., Solli P. (2015). Longitudinal studies. J. Thorac. Dis..

[131] Zelinski E.M., Burnight K.P. (1997). Sixteen-year longitudinal and time lag changes in memory and cognition in older adults. Psychol. Aging.

[132] Tombaugh T.N. (2004). Trail Making Test A and B: Normative data stratified by age and education. Arch. Clin. Neuropsychol..

[133] Sabia S., Kivimaki M., Shipley M.J., Marmot M.G., Singh-Manoux A. (2009). Body mass index over the adult life course and cognition in late midlife: The Whitehall II Cohort Study. Am. J. Clin. Nutr..

[134] Besser L.M., Gill D.P., Monsell S.E., Brenowitz W., Meranus D.H., Kukull W., Gustafson D.R. (2014). Body mass index, weight change, and clinical progression in mild cognitive impairment and Alzheimer disease. Alzheimer Dis. Assoc. Disord..

[135] Gu Y., Scarmeas N., Cosentino S., Brandt J., Albert M., Blacker D., Dubois B., Stern Y. (2014). Change in body mass index before and after Alzheimer’s disease onset. Curr. Alzheimer Res..

[136] Qizilbash N., Gregson J., Johnson M.E., Pearce N., Douglas I., Wing K., Evans S.J.W., Pocock S.J. (2015). BMI and risk of dementia in two million people over two decades: A retrospective cohort study. Lancet Diabetes Endocrinol.

[137] Fitzpatrick A.L., Kuller L.H., Lopez O.L., Diehr P., O’Meara E.S., Longstreth W.T., Luchsinger J.A. (2009). Midlife and late-life obesity and the risk of dementia: Cardiovascular health study. Arch. Neurol..

[138] Harrison J.K., Shenkin S.D. (2015). Body mass index and the risk of dementia--what do we know and what should we do. J. R. Coll. Physicians Edinb..

[139] Snijder M.B., Dekker J.M., Visser M., Bouter L.M., Stehouwer C.D., Yudkin J.S., Heine R.J., Nijpels G., Seidell J.C. (2004). Trunk fat and leg fat have independent and opposite associations with fasting and postload glucose levels: The Hoorn study. Diabetes Care.

[140] Chrousos G.P. (2009). Stress and disorders of the stress system. Nat. Rev. Endocrinol..

[141] McEwen B.S. (1998). Stress, adaptation, and disease. Allostasis and allostatic load. Ann. N. Y. Acad. Sci..

[142] McEwen B.S. (2004). Protection and damage from acute and chronic stress: Allostasis and allostatic overload and relevance to the pathophysiology of psychiatric disorders. Ann. N. Y. Acad. Sci..

[143] McEwen B.S. (2008). Central effects of stress hormones in health and disease: Understanding the protective and damaging effects of stress and stress mediators. Eur. J. Pharmacol..

[144] Kyrou I., Chrousos G.P., Tsigos C. (2006). Stress, visceral obesity, and metabolic complications. Ann. N. Y. Acad. Sci..

[145] Lupien S.J., Fiocco A., Wan N., Maheu F., Lord C., Schramek T., Tu M.T. (2005). Stress hormones and human memory function across the lifespan. Psychoneuroendocrinology.

[146] Sinha R., Jastreboff A.M. (2013). Stress as a common risk factor for obesity and addiction. Biol. Psychiatry.

[147] Rohleder N., Kirschbaum C. (2007). Effects of nutrition on neuro-endocrine stress responses. Curr. Opin. Clin. Nutr. Metab. Care.

[148] Maniam J., Morris M.J. (2012). The link between stress and feeding behaviour. Neuropharmacology.

[149] Picard M., Juster R.P., McEwen B.S. (2014). Mitochondrial allostatic load puts the ‘gluc’ back in glucocorticoids. Nat. Rev. Endocrinol..

[150] Marniemi J., Kronholm E., Aunola S., Toikka T., Mattlar C.E., Koskenvuo M., Rönnemaa T. (2002). Visceral fat and psychosocial stress in identical twins discordant for obesity. J. Intern. Med..

[151] Pyykkönen A.J., Räikkönen K., Tuomi T., Eriksson J.G., Groop L., Isomaa B. (2010). Stressful life events and the metabolic syndrome: The prevalence, prediction and prevention of diabetes (PPP)-Botnia Study. Diabetes Care.

[152] Brunner E.J., Chandola T., Marmot M.G. (2007). Prospective effect of job strain on general and central obesity in the Whitehall II Study. Am. J. Epidemiol..

[153] Oliver G., Wardle J., Gibson E.L. (2000). Stress and food choice: A laboratory study. Psychosom. Med..

[154] Steptoe A., Lipsey Z., Wardle J. (1998). Stress, hassles and variations in alcohol consumption, food choice and physical exercise: A diary study. Br. J. Health Psychol..

[155] Ng D.M., Jeffery R.W. (2003). Relationships between perceived stress and health behaviors in a sample of working adults. Health Psychol..

[156] Conner M., Fitter M., Fletcher W. (1999). Stress and snacking: A diary study of daily hassles and between-meal snacking. Psychol. Health.

[157] Oliver G., Wardle J. (1999). Perceived effects of stress on food choice. Physiol. Behav..

[158] Gluck M.E., Geliebter A., Hung J., Yahav E. (2004). Cortisol, hunger, and desire to binge eat following a cold stress test in obese women with binge eating disorder. Psychosom. Med..

[159] O’Connor D.B., Jones F., Conner M., McMillan B., Ferguson E. (2008). Effects of daily hassles and eating style on eating behavior. Health Psychol..

[160] Björntorp P., Rosmond R. (2000). Obesity and cortisol. Nutrition.

[161] Rosmond R., Björntorp P. (2000). Occupational status, cortisol secretory pattern, and visceral obesity in middle-aged men. Obes. Res..

[162] Epel E.S., McEwen B., Seeman T., Matthews K., Castellazzo G., Brownell K.D., Bell J., Ickovics J.R. (2000). Stress and body shape: Stress-induced cortisol secretion is consistently greater among women with central fat. Psychosom. Med..

[163] Picard M., Turnbull D.M. (2013). Linking the metabolic state and mitochondrial DNA in chronic disease, health, and aging. Diabetes.

[164] Wolf O.T. (2009). Stress and memory in humans: Twelve years of progress. Brain Res..

[165] Lupien S.J., Maheu F., Tu M., Fiocco A., Schramek T.E. (2007). The effects of stress and stress hormones on human cognition: Implications for the field of brain and cognition. Brain Cogn..

[166] Lupien S.J., de Leon M., de Santi S., Convit A., Tarshish C., Nair N.P., Thakur M., McEwen B.S., Hauger R.L., Meaney M.J. (1998). Cortisol levels during human aging predict hippocampal atrophy and memory deficits. Nat. Neurosci..

[167] Dai J., Buijs R., Swaab D. (2004). Glucocorticoid hormone (cortisol) affects axonal transport in human cortex neurons but shows resistance in Alzheimer’s disease. Br. J. Pharmacol..

[168] Lasikiewicz N., Hendrickx H., Talbot D., Dye L. (2013). Exploring stress-induced cognitive impairment in middle aged, centrally obese adults. Stress.

[169] Battaglia M.R., Di Fazio C., Battaglia S. (2023). Activated Tryptophan-Kynurenine metabolic system in the human brain is associated with learned fear. Front. Mol. Neurosci..

[170] Polyák H., Galla Z., Nánási N., Cseh E.K., Rajda C., Veres G., Spekker E., Szabó Á., Klivényi P., Tanaka M. (2023). The Tryptophan-Kynurenine Metabolic System Is Suppressed in Cuprizone-Induced Model of Demyelination Simulating Progressive Multiple Sclerosis. Biomedicines.

[171] Dye L., Boyle N.B., Champ C., Lawton C. (2017). The relationship between obesity and cognitive health and decline. Proc. Nutr. Soc..

[172] Hoyland A., Lawton C.L., Dye L. (2008). Acute effects of macronutrient manipulations on cognitive test performance in healthy young adults: A systematic research review. Neurosci. Biobehav. Rev..

[173] Lamport D., Dye L., Wightman J., Lawton C.L. (2012). The effects of flavonoid and other polyphenol consumption on cognitive performance: A systematic research review of human experimental and epidemiological studies. Nutr. Aging.

[174] Jacobs D.R., Gross M.D., Tapsell L.C. (2009). Food synergy: An operational concept for understanding nutrition. Am. J. Clin. Nutr..

[175] Nilsson A., Tovar J., Johansson M., Radeborg K., Björck I. (2013). A diet based on multiple functional concepts improves cognitive performance in healthy subjects. Nutr. Metab..

[176] Smith P.J., Blumenthal J.A., Babyak M.A., Craighead L., Welsh-Bohmer K.A., Browndyke J.N., Strauman T.A., Sherwood A. (2010). Effects of the dietary approaches to stop hypertension diet, exercise, and caloric restriction on neurocognition in overweight adults with high blood pressure. Hypertension.

[177] Sofi F., Macchi C., Abbate R., Gensini G.F., Casini A. (2014). Mediterranean diet and health status: An updated meta-analysis and a proposal for a literature-based adherence score. Public Health Nutr..

[178] National Institute for Health and Care Excellence (2014). Maintaining a Healthy Weight and Preventing Excess Weight Gain among Children and Adults: Draft Guideline. http://www.nice.org.uk/guidance/gid-phg78/resources/maintaining-a-healthy-weight-and-preventingexcess-weight-gain-among-children-and-adults-draftguideline2.

[179] Committee of the European Union (2014). Council Conclusionson Nutrition and Physical Activity. http://www.consilium.europa.eu/uedocs/cms_data/docs/pressdata/en/lsa/143285.pdf.

[180] Tangney C.C., Kwasny M.J., Li H., Wilson R.S., Evans D.A., Morris M.C. (2011). Adherence to a Mediterranean-type dietary pattern and cognitive decline in a community population. Am. J. Clin. Nutr..

[181] Wengreen H., Munger R.G., Cutler A., Quach A., Bowles A., Corcoran C., Tschanz J.T., Norton M.C., Welsh-Bohmer K.A. (2013). Prospective study of Dietary Approaches to Stop Hypertension- and Mediterranean-style dietary patterns and age-related cognitive change: The Cache County Study on Memory, Health and Aging. Am. J. Clin. Nutr..

[182] Féart C., Samieri C., Rondeau V., Amieva H., Portet F., Dartigues J.F., Scarmeas N., Barberger-Gateau P. (2009). Adherence to a Mediterranean diet, cognitive decline, and risk of dementia. JAMA.

[183] Scarmeas N., Stern Y., Mayeux R., Manly J.J., Schupf N., Luchsinger J.A. (2009). Mediterranean diet and mild cognitive impairment. Arch. Neurol..

[184] Tsivgoulis G., Judd S., Letter A.J., Alexandrov A.V., Howard G., Nahab F., Unverzagt F.W., Moy C., Howard V.J., Kissela B. (2013). Adherence to a Mediterranean diet and risk of incident cognitive impairment. Neurology.

[185] Samieri C., Grodstein F., Rosner B.A., Kang J.H., Cook N.R., Manson J.E., Buring J.E., Willett W.C., Okereke O.I. (2013). Mediterranean diet and cognitive function in older age. Epidemiology.

[186] Ye X., Scott T., Gao X., Maras J.E., Bakun P.J., Tucker K.L. (2013). Mediterranean diet, healthy eating index 2005, and cognitive function in middle-aged and older Puerto Rican adults. J. Acad. Nutr. Diet..

[187] Gardener S., Gu Y., Rainey-Smith S.R., Keogh J.B., Clifton P.M., Mathieson S.L., Taddei K., Mondal A., Ward V.K., Scarmeas N. (2012). Adherence to a Mediterranean diet and Alzheimer’s disease risk in an Australian population. Transl. Psychiatry.

[188] Scarmeas N., Stern Y., Mayeux R., Luchsinger J.A. (2006). Mediterranean diet, Alzheimer disease, and vascular mediation. Arch. Neurol..

[189] Singh B., Parsaik A.K., Mielke M.M., Erwin P.J., Knopman D.S., Petersen R.C., Roberts R.O. (2014). Association of mediterranean diet with mild cognitive impairment and Alzheimer’s disease: A systematic review and meta-analysis. J. Alzheimers Dis..

[190] Psaltopoulou T., Sergentanis T.N., Panagiotakos D.B., Sergentanis I.N., Kosti R., Scarmeas N. (2013). Mediterranean diet, stroke, cognitive impairment, and depression: A meta-analysis. Ann. Neurol..

[191] Sofi F., Abbate R., Gensini G.F., Casini A. (2010). Accruing evidence on benefits of adherence to the Mediterranean diet on health: An updated systematic review and meta-analysis. Am. J. Clin. Nutr..

[192] Kesse-Guyot E., Andreeva V.A., Lassale C., Ferry M., Jeandel C., Hercberg S., Galan P. (2013). Mediterranean diet and cognitive function: A French study. Am. J. Clin. Nutr..

[193] Cherbuin N., Anstey K.J. (2012). The Mediterranean diet is not related to cognitive change in a large prospective investigation: The PATH Through Life study. Am. J. Geriatr. Psychiatry.

[194] Valls-Pedret C., Sala-Vila A., Serra-Mir M., Corella D., de la Torre R., Martínez-González M.Á., Martínez-Lapiscina E.H., Fitó M., Pérez-Heras A., Salas-Salvadó J. (2015). Mediterranean Diet and Age-Related Cognitive Decline: A Randomized Clinical Trial. JAMA Intern. Med..

[195] Spitznagel M.B., Hawkins M., Alosco M., Galioto R., Garcia S., Miller L., Gunstad J. (2015). Neurocognitive Effects of Obesity and Bariatric Surgery. Eur. Eat. Disord Rev..

[196] Alosco M.L., Spitznagel M.B., Strain G., Devlin M., Crosby R.D., Mitchell J.E., Gunstad J. (2014). Family history of Alzheimer’s disease limits improvement in cognitive function after bariatric surgery. SAGE Open Med..

[197] Li C.M., Song J.R., Zhao J., Wang C.F., Zhang C.S., Wang H.D., Zhang Q., Liu D.F., Ma Z.Y., Yuan J.H. (2022). The effects of bariatric surgery on cognition in patients with obesity: A systematic review and meta-analysis. Surg. Obes. Relat. Dis..

[198] Jackson A.S., Stanforth P.R., Gagnon J., Rankinen T., Leon A.S., Rao D.C., Skinner J.S., Bouchard C., Wilmore J.H. (2002). The effect of sex, age and race on estimating percentage body fat from body mass index: The Heritage Family Study. Int. J. Obes. Relat. Metab. Disord..

[199] Profenno L.A., Porsteinsson A.P., Faraone S.V. (2010). Meta-analysis of Alzheimer’s disease risk with obesity, diabetes, and related disorders. Biol. Psychiatry.

[200] Gudala K., Bansal D., Schifano F., Bhansali A. (2013). Diabetes mellitus and risk of dementia: A meta-analysis of prospective observational studies. J. Diabetes. Investig..

[201] Norgan N.G. (1994). Population differences in body composition in relation to the body mass index. Eur. J. Clin. Nutr..

[202] Solomons N.W., Kumanyika S. (2000). Implications of racial distinctions for body composition and its diagnostic assessment. Am. J. Clin. Nutr..

[203] Jeong S.M., Lee D.H., Rezende L.F.M., Giovannucci E.L. (2023). Different correlation of body mass index with body fatness and obesity-related biomarker according to age, sex and race-ethnicity. Sci. Rep..

[204] Greenland S., Robins J.M. (1986). Identifiability, exchangeability, and epidemiological confounding. Int. J. Epidemiol..

[205] Greenland S., Morgenstern H. (2001). Confounding in health research. Annu. Rev. Public Health.

[206] Grimes D.A., Schulz K.F. (2002). Bias and causal associations in observational research. Lancet.

[207] Vandenbroucke J.P., von Elm E., Altman D.G., Gøtzsche P.C., Mulrow C.D., Pocock S.J., Poole C., Schlesselman J.J., Egger M. (2007). Strengthening the Reporting of Observational Studies in Epidemiology (STROBE): Explanation and elaboration. PLoS Med..

[208] Anstey K.J., Cherbuin N., Budge M., Young J. (2011). Body mass index in midlife and late-life as a risk factor for dementia: A meta-analysis of prospective studies. Obes. Rev..

[209] Gorospe E.C., Dave J.K. (2007). The risk of dementia with increased body mass index. Age Ageing.

[210] Livingston G., Huntley J., Sommerlad A., Ames D., Ballard C., Banerjee S., Brayne C., Burns A., Cohen-Mansfield J., Cooper C. (2020). Dementia prevention, intervention, and care: 2020 report of the Lancet Commission. Lancet.

[211] Yates K.F., Sweat V., Yau P.L., Turchiano M.M., Convit A. (2012). Impact of metabolic syndrome on cognition and brain: A selected review of the literature. Arter. Thromb. Vasc. Biol..

[212] Batty G.D., Deary I.J., Gottfredson L.S. (2007). Premorbid (early life) IQ and later mortality risk: Systematic review. Ann. Epidemiol..

[213] Bruce-Keller A.J., Keller J.N., Morrison C.D. (2009). Obesity and vulnerability of the CNS. Biochim. Biophys. Acta.

[214] Siervo M., Arnold R., Wells J.C., Tagliabue A., Colantuoni A., Albanese E., Brayne C., Stephan B.C. (2011). Intentional weight loss in overweight and obese individuals and cognitive function: A systematic review and meta-analysis. Obes. Rev..

[215] Tran K.N., Nguyen N.P.K., Nguyen L.T.H., Shin H.M., Yang I.J. (2023). Screening for Neuroprotective and Rapid Antidepressant-like Effects of 20 Essential Oils. Biomedicines.

